# Supplementary material for: Fgf signalling triggers an intrinsic mesodermal timer that determines the duration of limb patterning
Source: Nat Commun. 2023 Sep 20;14:5841. doi: 10.1038/s41467-023-41457-6 (PMC10511490; doi:10.1038/s41467-023-41457-6)
Supplement: Supplementary file 1 — Supplementary Information [file 41467_2023_41457_MOESM1_ESM.pdf]

Supplementary information: Fgf signalling triggers an intrinsic mesodermal timer that determines the duration of limb patterning

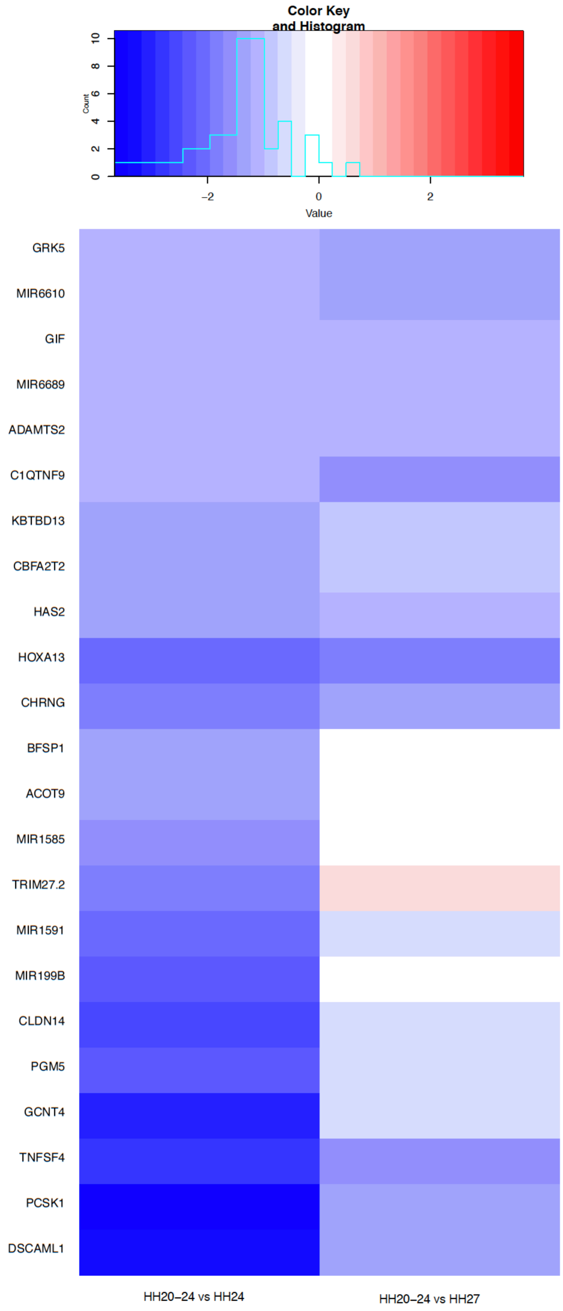

Supplementary Figure 1 - Intrinsically expressed genes in Cluster 1

23 genes including *Hoxa13* generally show much lower expression in stage HH20-24 grafts (HH24g) after 24h compared with donor stage HH24 tissue and also host stage tissue HH27.

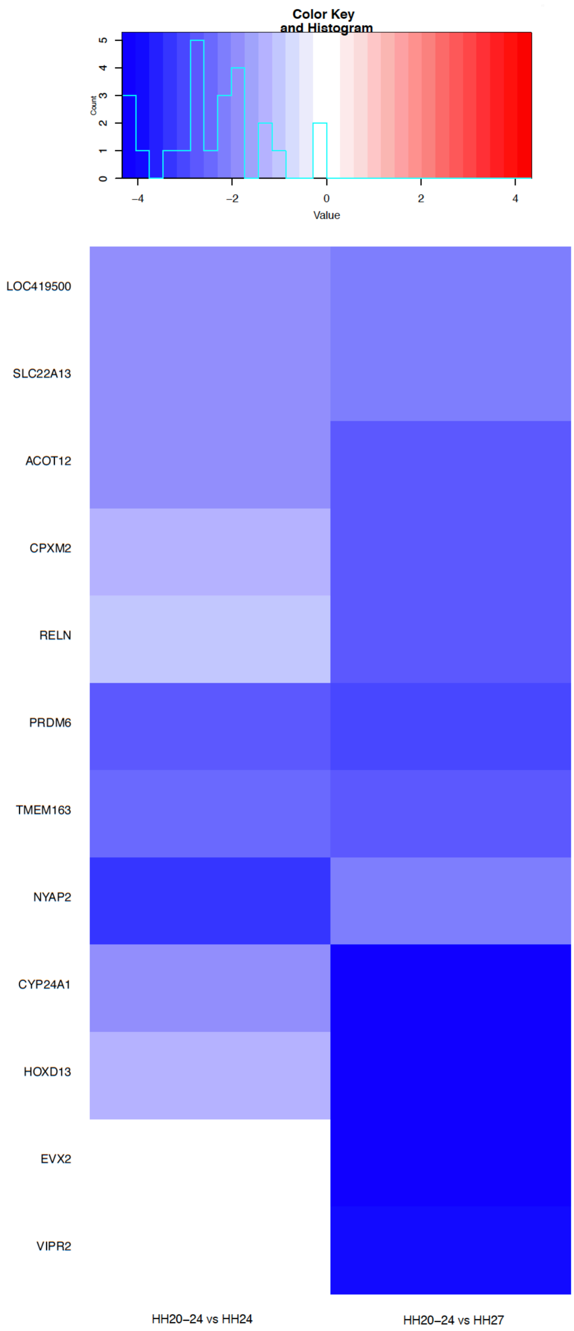

Supplementary Figure 2 - Intrinsically expressed genes in Cluster 2

12 genes including *Hoxd13* generally show much lower expression in stage HH20-24 grafts (HH24g) after 24h compared with donor stage HH24 tissue and also host stage tissue HH27.

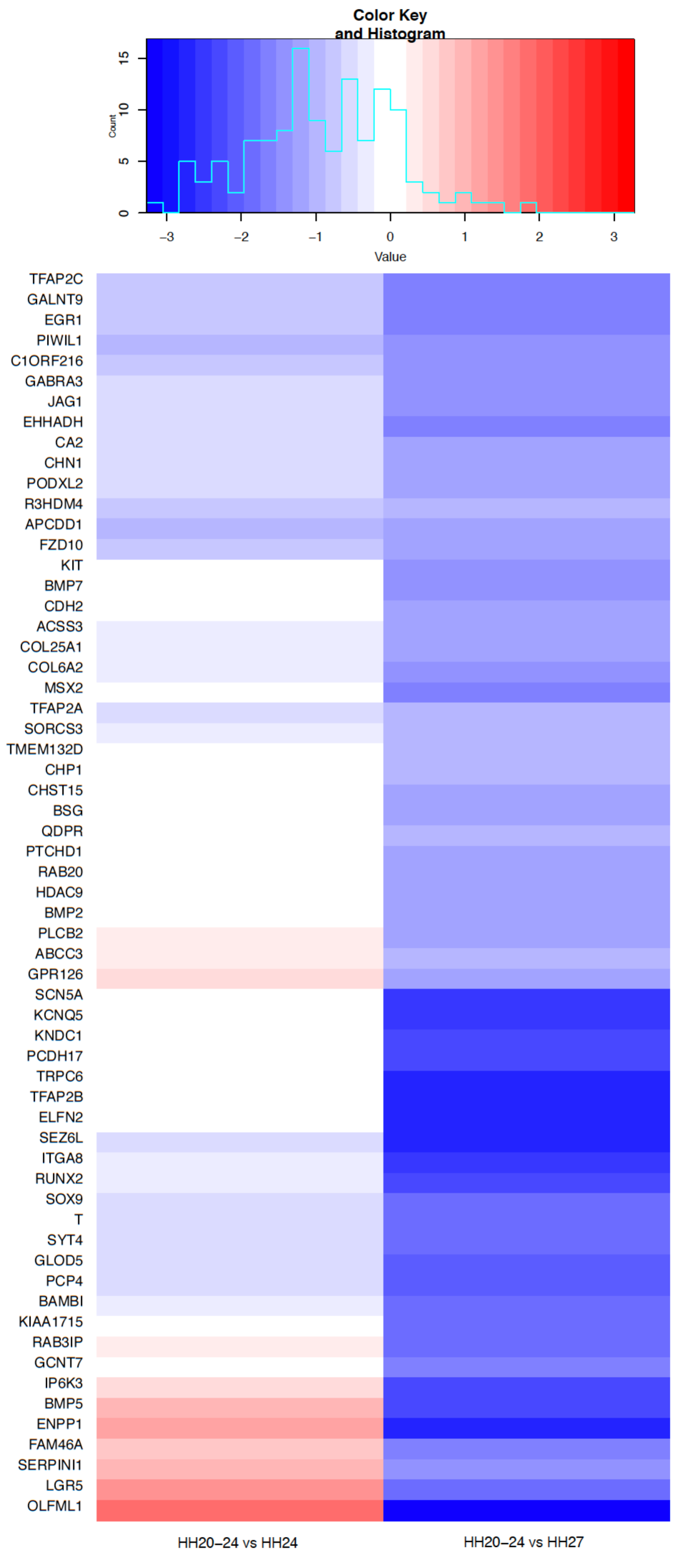

Supplementary Figure 3 - Intrinsically expressed genes in Cluster 3

61 genes including several associated with Bmp signalling and differentiation generally show similar levels of expression in stage HH20-24 grafts (HH24g) after 24h compared with donor stage HH24 tissue, and much lower expression compared with host stage tissue HH27.

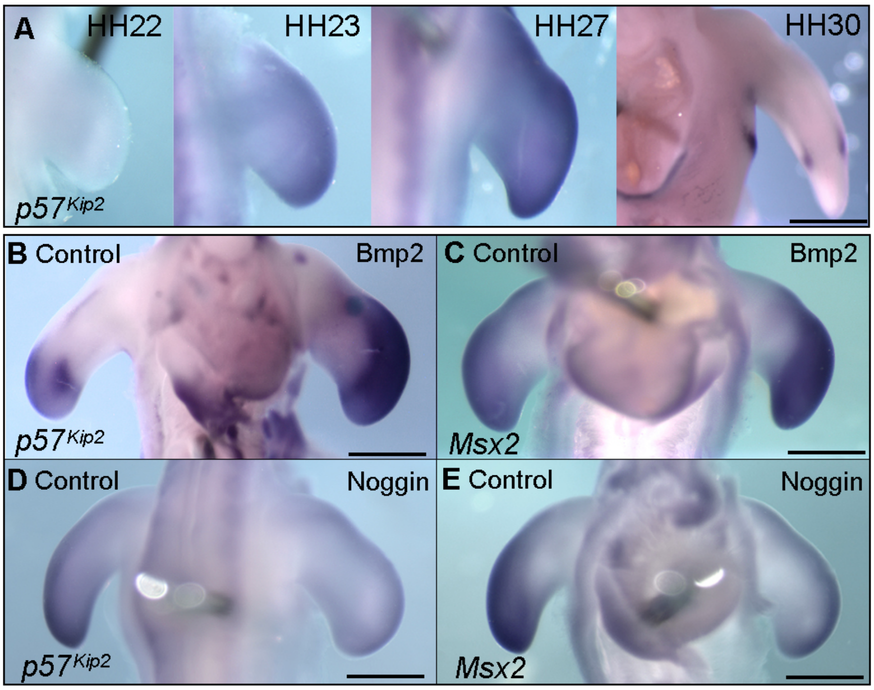

Supplementary Figure 4 - Expression and Bmp2/Noggin regulation of *p57^kip2^*

a) *p57^kip^* is expressed in chick wing distal mesoderm between HH23 and HH30, at which stage, it is only detectable in dorsal and ventral muscle masses (*n*=>3 for all stages). **b**) Bmp2-soaked beads implanted into the distal mesoderm of HH24 wing buds up-regulate *p57^kip2^* expression 24h later at HH27 (*n*=4/4) and **c**) *Msx2* – a transcriptional target of Bmp signalling (*n*=3/3). **d**) Noggin-soaked beads implanted into the distal mesoderm of HH24 wing buds down-regulate *p57^kip2^* expression 24h later at HH27 (*n*=3/3) and **e**) *Msx2* (*n*=3/3). Scale bars - 500μm.

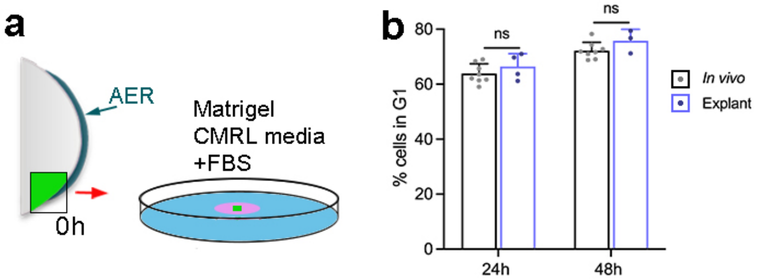

Supplementary Figure 5 - A chick wing explant system

a) The posterior-distal region of the wing bud including the AER and the polarising region is dissected at HH20 (designated as 0h) and cultured in Matrigel, CMRL media and FBS. **b**) Flow cytometric analyses at 24h (n=8, 4, p=0.32) and 48h (n=8, 3, p=0.50) reveals no significant difference in the percentage of cells in G1-phase in explants and in equivalent *in vivo* tissue: two-tailed unpaired *t*-tests were carried out at each time-point. n= biologically independent samples. All bars represent the mean +/- standard deviation represented as error bars. Individual data points are presented as dots overlaid within each bar. Statistical significance was determined through two-tailed unpaired *t*-tests. Source data are provided as a Source Data file.

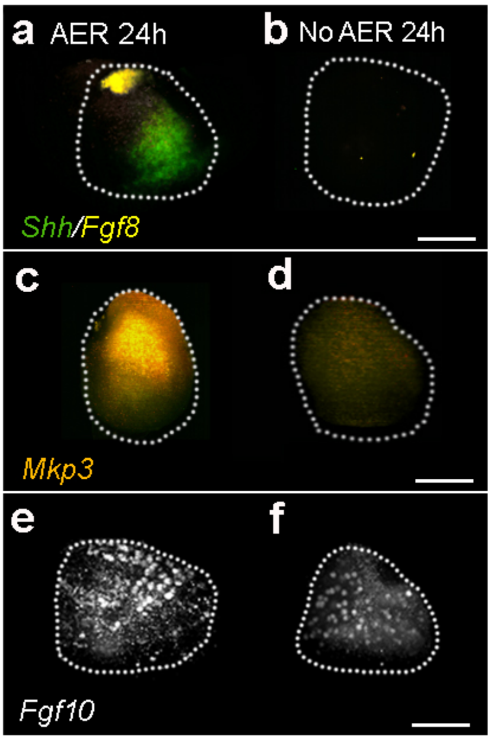

Supplementary Figure 6 - Gene expression analyses in explants cultured without an AER

a, **b**) *Shh* and *Fgf8* are undetectable 24h after AER removal (*n*=4/4). **c**, **d**) *Mkp3* is down-regulated 24h after AER removal (*n*=3/3). **e**, **f**) *Fgf10* is down-regulated 24h after AER removal (*n*=3/3). Scale bars - 200μm.

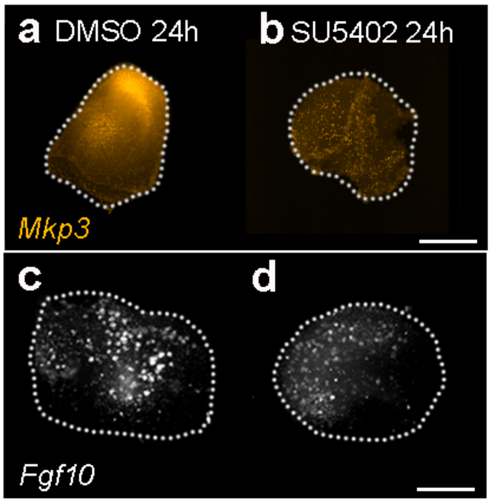

Supplementary Figure 7 - Gene expression analyses in explants treated with SU5402

a, **b**) *Mkp3* is down-regulated 24h after AER removal (*n*=8/8). **c**, **d**) *Fgf10* is down-regulated 24h after AER removal (*n*=4/4). Scale bars - 200μm.

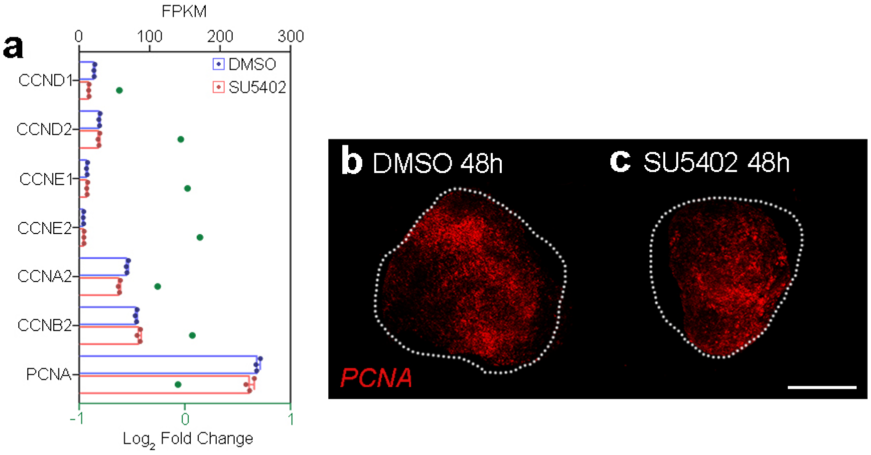

Supplementary Figure 8 - Expression of cell cycle regulators in explants treated with SU5402

a) Expression of cell cycle regulators in control DMSO- and SU5402-treated explants at 48h as shown by log_2-fold changes (*n*=3; <2-fold change; differential expression analysis was performed using DESeq2 and the resulting *p*-values were adjusted using the Benjamini and Hochberg procedure for controlling the false discovery rate; adjusted *p*-value of <0.05; green dots) and normalised read-counts mapped to each gene (FKPM – Fragments of Kilobase of Exon Per Million Mapped Fragment). **b**) *PCNA* is expressed in control (*n*=3/3) and **c**) in SU5402 (*n*=4/4) explants at 48h. Bars represent the mean +/- standard deviation represented as error bars. Individual data points are presented as dots overlaid within each bar. Source data are provided as a Source Data file with individual *p*-values. Scale bars - 200μM.
