# Supplementary material for: Fgf signalling triggers an intrinsic mesodermal timer that determines the duration of limb patterning
Source: Nat Commun. 2023 Sep 20;14:5841. doi: 10.1038/s41467-023-41457-6 (PMC10511490; doi:10.1038/s41467-023-41457-6)
Supplement: Supplementary file 3 — Description of Additional Supplementary Files [file 41467_2023_41457_MOESM3_ESM.pdf]

Description of Additional Supplementary Files

File Name: Supplementary Data 1
Description: Differentially expressed genes HH24 vs. HH24g

File Name: Supplementary Data 2
Description: Differentially expressed genes HH24g vs. HH27

File Name: Supplementary Data 3
Description: Differentially expressed genes SU5402 vs. DMSO

File Name: Supplementary Data 4
Description: All genes SU5402 vs. DMSO

File Name: Supplementary Data 5
Description: HCR probe accession numbers

File Name: Supplementary Data 6
Description: Flow cytometry gating information – Figure 3, S5
