# Supplementary material for: Fgf signalling triggers an intrinsic mesodermal timer that determines the duration of limb patterning
Source: Nat Commun. 2023 Sep 20;14:5841. doi: 10.1038/s41467-023-41457-6 (PMC10511490; doi:10.1038/s41467-023-41457-6)
Supplement: Supplementary file 9 — Supplementary Data 6 [file 41467_2023_41457_MOESM9_ESM.pdf]

Supplementary Figure 5b - In vivo 24h - G1 = 61.38

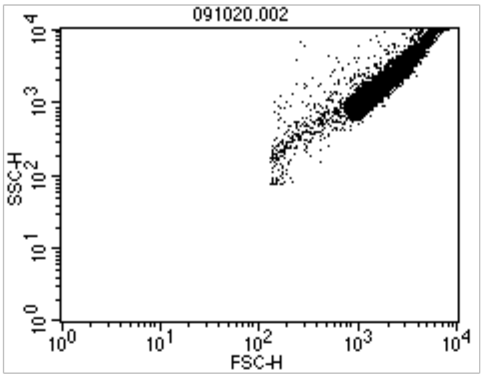

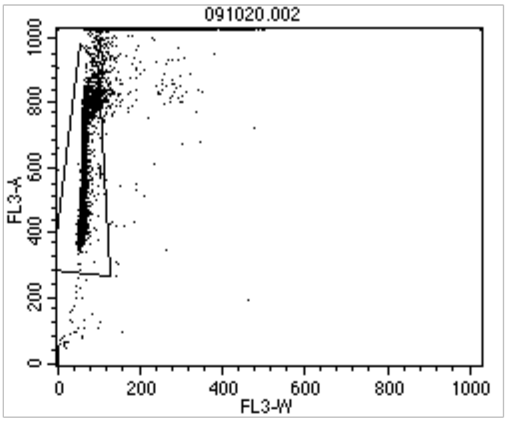

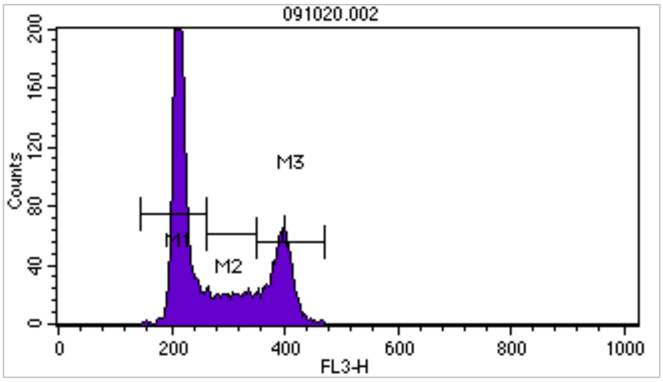

Marker	Left, Right	Events	% Gated	% Total	Mean	Geo Mean	CV	Median	Peak Ch
All	0, 1023	10000	100.00	80.57	272.50	262.69	28.27	225.00	212
M1	145, 262	6138	61.38	49.45	216.43	216.02	6.25	214.00	212
M2	262, 350	1386	13.86	11.17	306.20	305.08	8.54	307.00	266
M3	350, 471	2511	25.11	20.23	391.49	391.00	5.02	393.00	397

Supplementary Fig 5b - In vivo 24h - G1 = 59.03

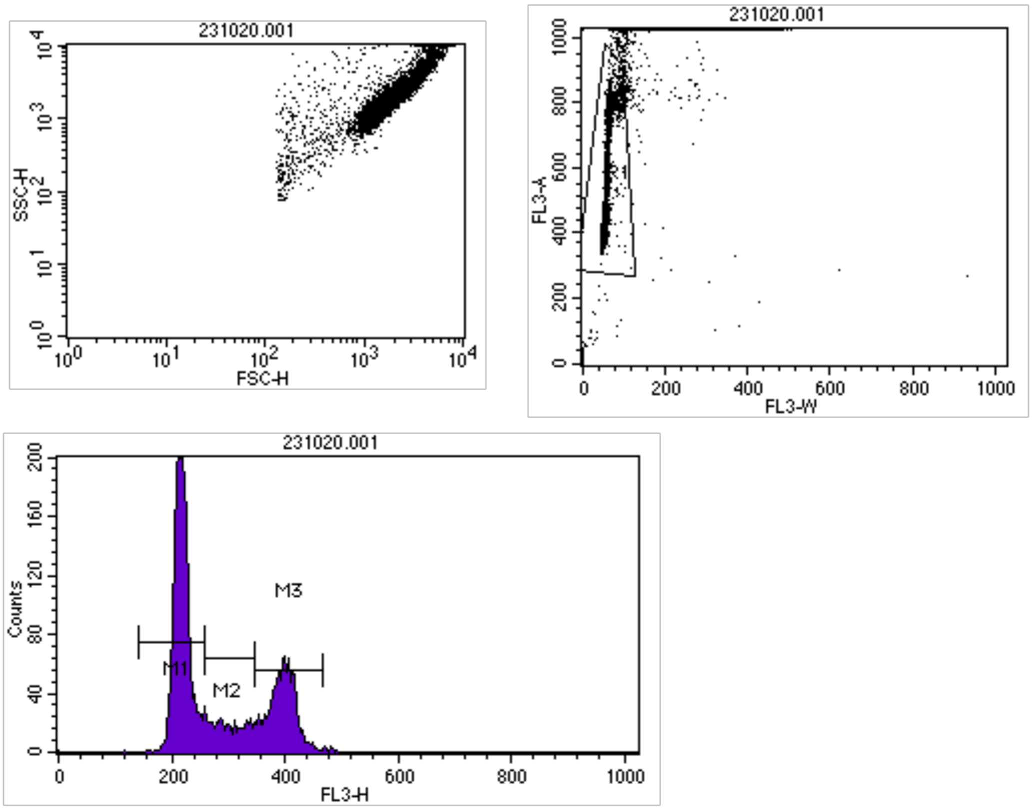

Marker	Left, Right	Events	% Gated	% Total	Mean	Geo Mean	CV	Median	Peak Ch
All	0, 1023	10000	100.00	82.59	277.95	267.54	28.53	229.00	216
M1	142, 258	5903	59.03	48.75	217.83	217.46	5.82	217.00	216
M2	258, 347	1320	13.20	10.90	301.78	300.61	8.80	301.00	259
M3	347, 467	2790	27.90	23.04	393.57	393.01	5.32	395.00	399

Supplementary Figure 5b - In vivo 24h – G1 = 62.13

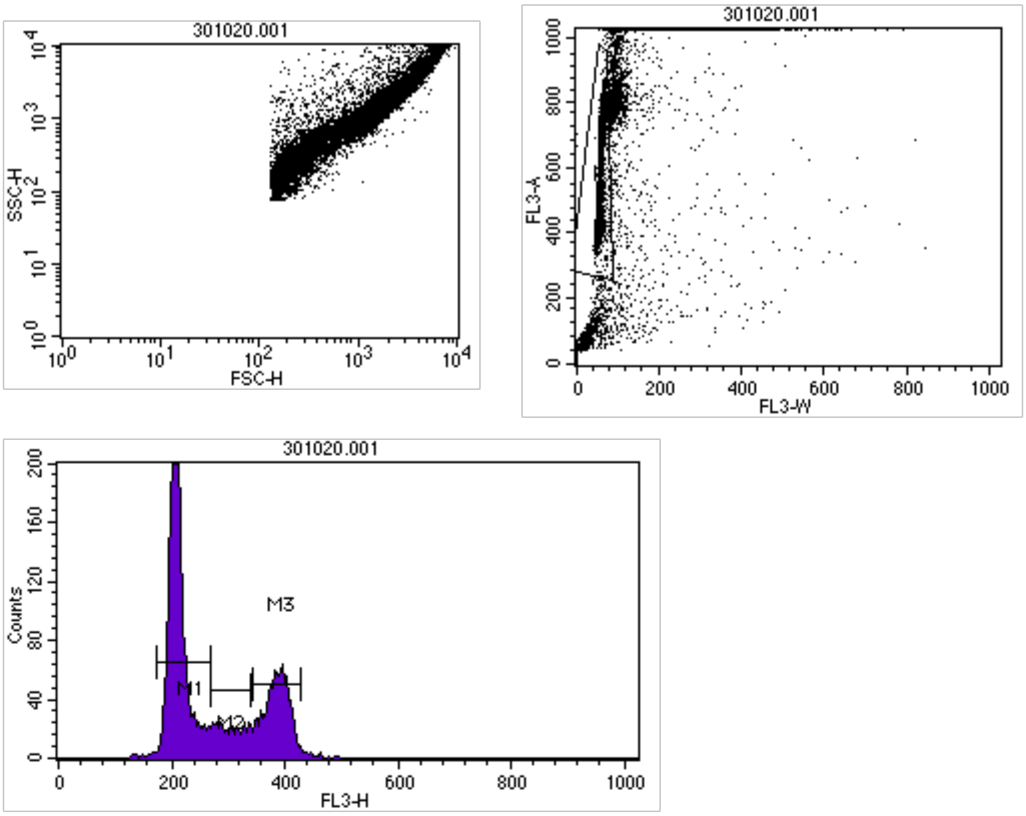

Marker	Left, Right	Events	% Gated	% Total	Mean	Geo Mean	CV	Median	Peak Ch
All	0, 1023	10000	100.00	52.12	268.49	258.02	29.25	221.00	206
M1	173, 269	6153	61.53	32.07	211.70	211.05	8.06	208.00	206
M2	269, 340	1116	11.16	5.82	304.15	303.42	6.94	304.00	273
M3	343, 428	2634	26.34	13.73	383.69	383.20	5.06	385.00	395

Supplementary Figure 5b - In vivo 24h – G1 = 61.66

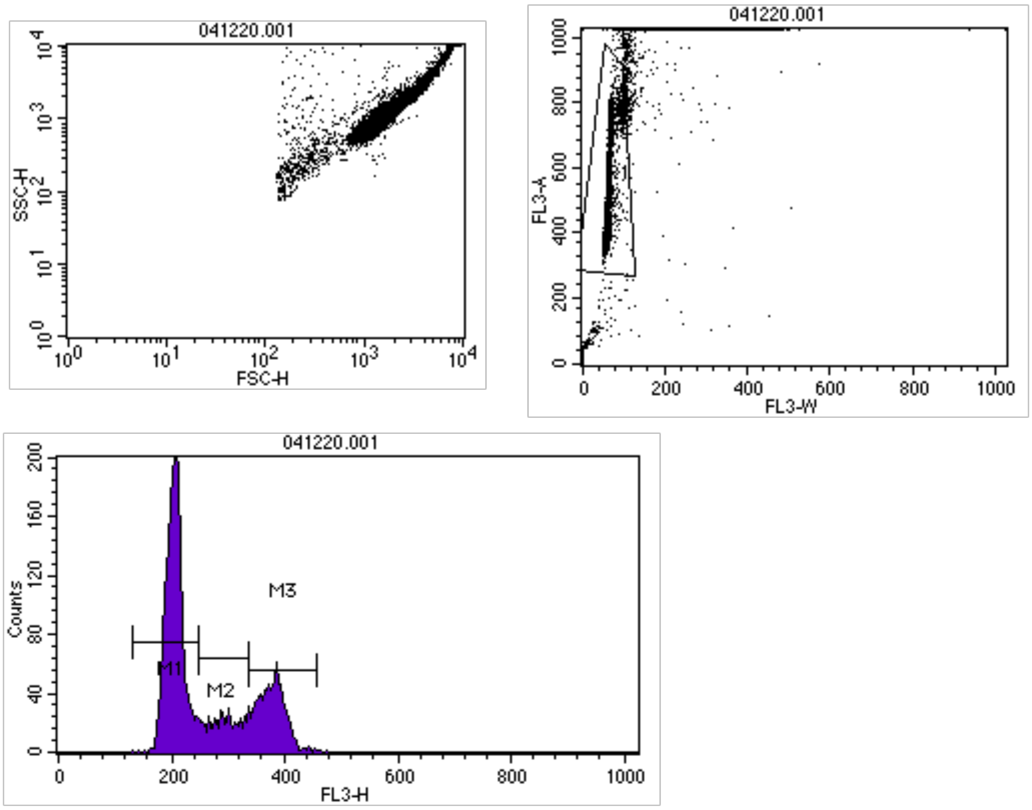

Marker	Left, Right	Events	% Gated	% Total	Mean	Geo Mean	CV	Median	Peak Ch
All	0, 1023	10000	100.00	81.86	258.82	249.21	28.73	215.00	209
M1	131, 248	6166	61.66	50.47	205.29	204.80	6.97	205.00	209
M2	248, 336	1432	14.32	11.72	293.16	291.97	8.97	293.00	335
M3	336, 457	2430	24.30	19.89	374.61	374.05	5.48	375.00	383

Supplementary Figure 5b - In vivo 24h – G1 = 65.73

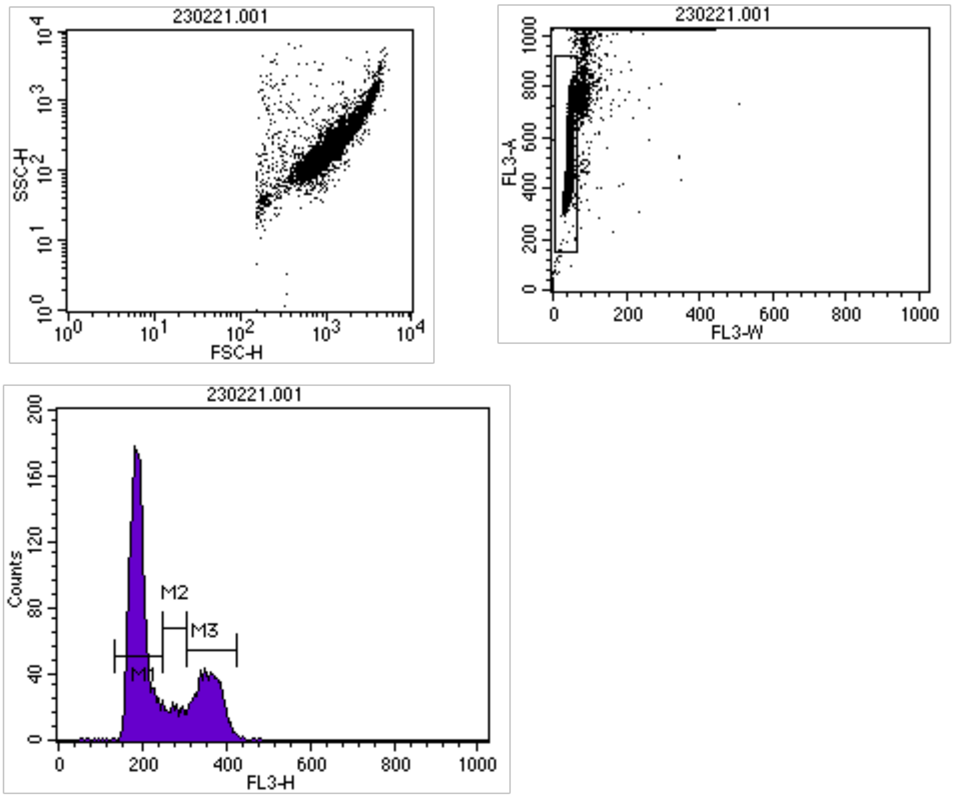

Marker	% Gated	% Total
All	100.00	79.28
M1	65.73	52.11
M2	8.65	6.86
M3	25.63	20.32

Supplementary Figure 5b - In vivo 24h – G1 = 67.96

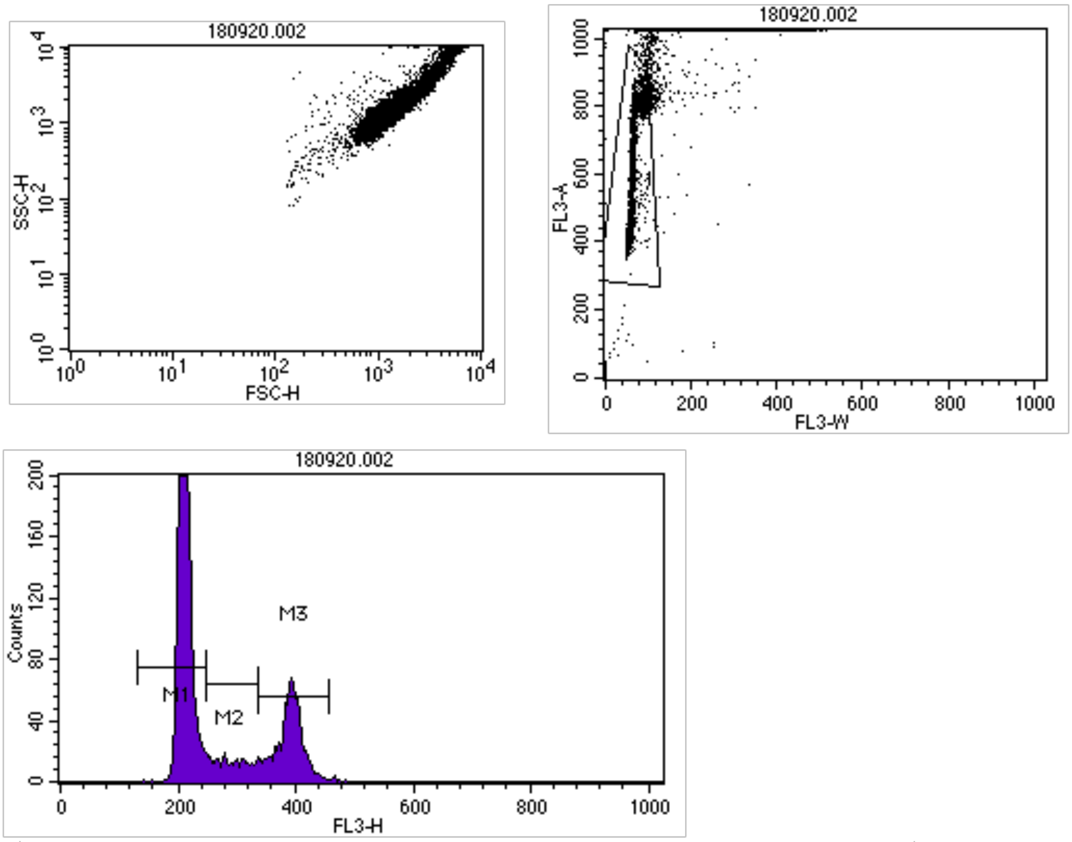

Marker	Left, Right	Events	% Gated	% Total	Mean	Geo Mean	CV	Median	Peak Ch
All	0, 1023	10000	100.00	77.89	259.69	250.26	29.16	216.00	207
M1	131, 248	6796	67.96	52.93	211.61	211.38	4.70	210.00	207
M2	248, 336	888	8.88	6.92	290.86	289.66	9.09	290.00	277
M3	336, 457	2331	23.31	18.16	387.72	387.15	5.36	390.00	392

Supplementary Figure 5b - In vivo 24h – G1 = 62.99

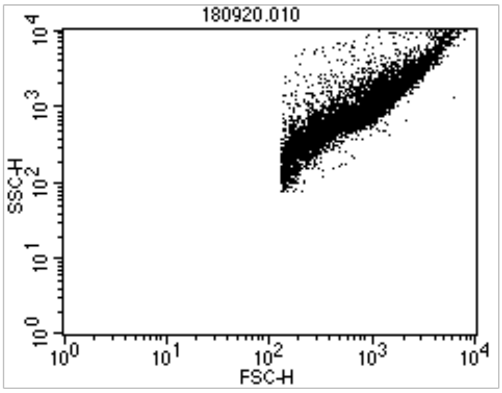

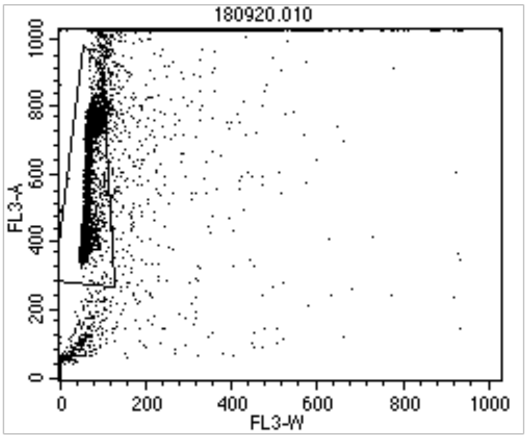

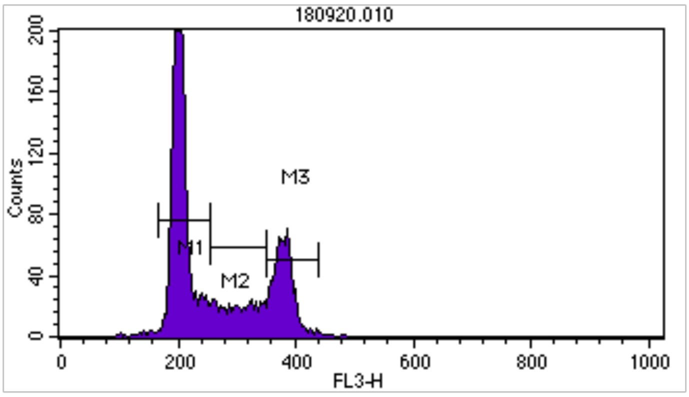

Marker	Left, Right	Events	% Gated	% Total	Mean	Geo Mean	CV	Median	Peak Ch
All	0, 1023	10000	100.00	70.99	257.02	247.13	29.31	210.00	204
M1	166, 255	6299	62.99	44.71	204.52	204.06	6.94	202.00	204
M2	255, 350	1492	14.92	10.59	303.57	302.25	9.28	304.00	258
M3	350, 439	2170	21.70	15.40	378.30	378.01	3.97	378.00	383

Supplementary Figure 5b - In vivo 24h – G1 = 63.57 (Summary of 2 readings – 62.44 (002) & 64.70 (012))

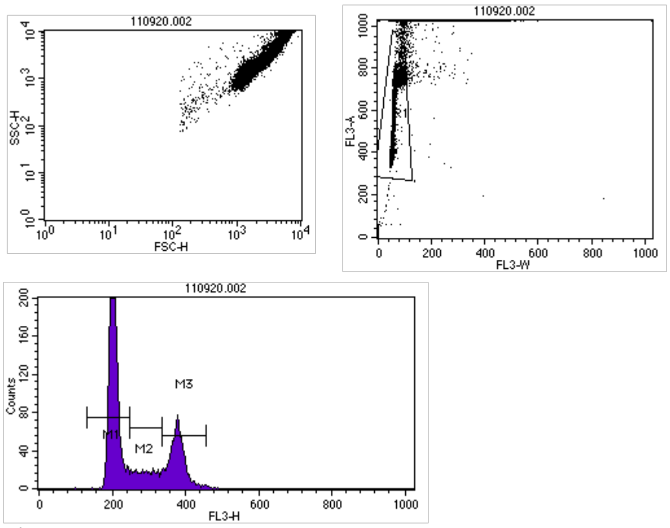

Marker	Left, Right	Events	% Gated	% Total	Mean	Geo Mean	CV	Median	Peak Ch
All	0, 1023	10000	100.00	75.74	260.31	250.18	29.53	213.00	201
M1	131, 248	6244	62.44	47.29	205.18	204.84	5.85	203.00	201
M2	248, 336	1153	11.53	8.73	292.94	291.77	8.92	293.00	310
M3	336, 457	2617	26.17	19.82	376.82	376.28	5.44	376.00	378

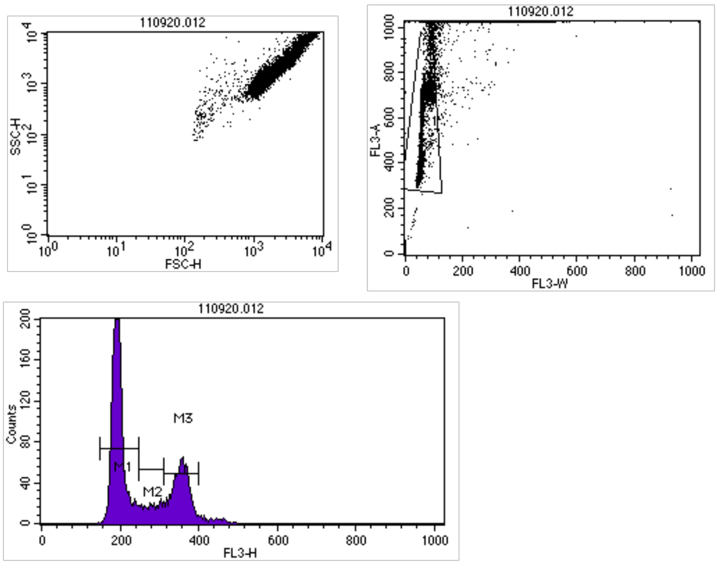

Marker	Left, Right	Events	% Gated	% Total	Mean	Geo Mean	CV	Median	Peak Ch
All	0, 1023	10000	100.00	78.49	246.12	236.20	30.21	202.00	193
M1	149, 248	6470	64.70	50.78	195.11	194.59	7.50	193.00	193
M2	248, 312	868	8.68	6.81	280.38	279.74	6.76	281.00	302
M3	312, 400	2529	25.29	19.85	353.56	353.04	5.41	355.00	359

Supplementary Figure 5b - In vivo 48h – G1 = 71.85

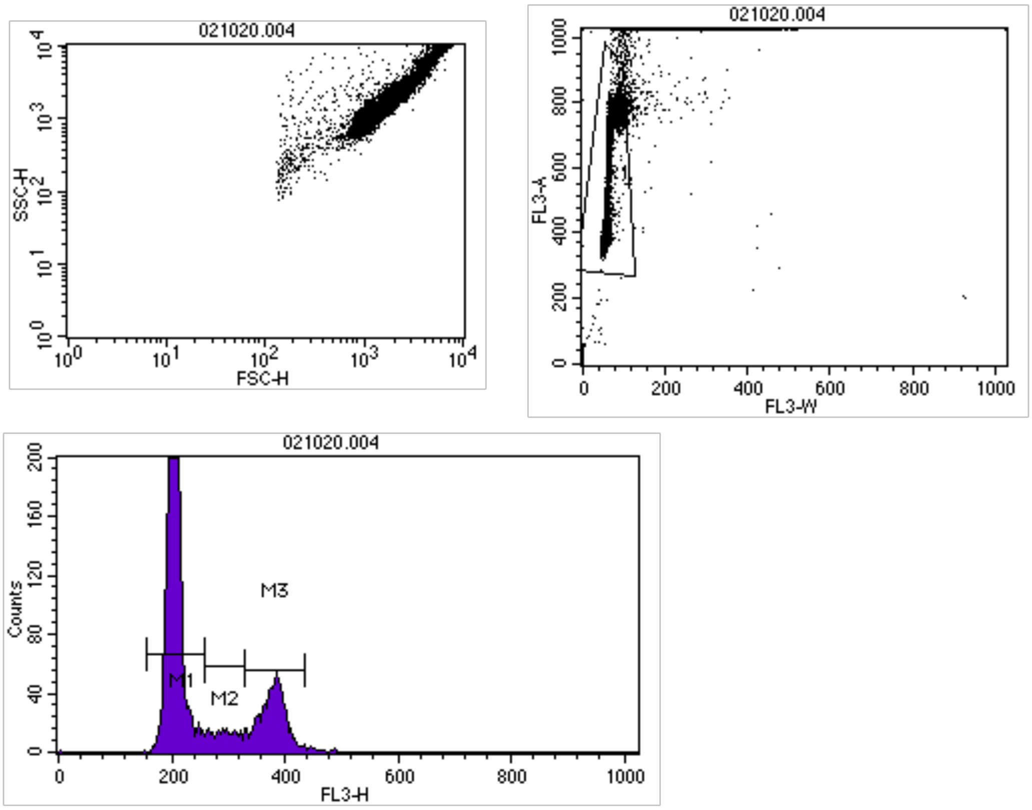

Marker	Left, Right	Events	% Gated	% Total	Mean	Geo Mean	CV	Median	Peak Ch
All	0, 1023	10000	100.00	79.55	247.97	238.88	29.34	209.00	205
M1	156, 258	7185	71.85	57.16	205.23	204.86	6.08	204.00	205
M2	258, 329	686	6.86	5.46	293.73	292.99	7.08	293.50	296
M3	329, 435	2124	21.24	16.90	376.07	375.48	5.59	378.00	384

Supplementary Figure 5b - In vivo 48h – G1 = 72.77

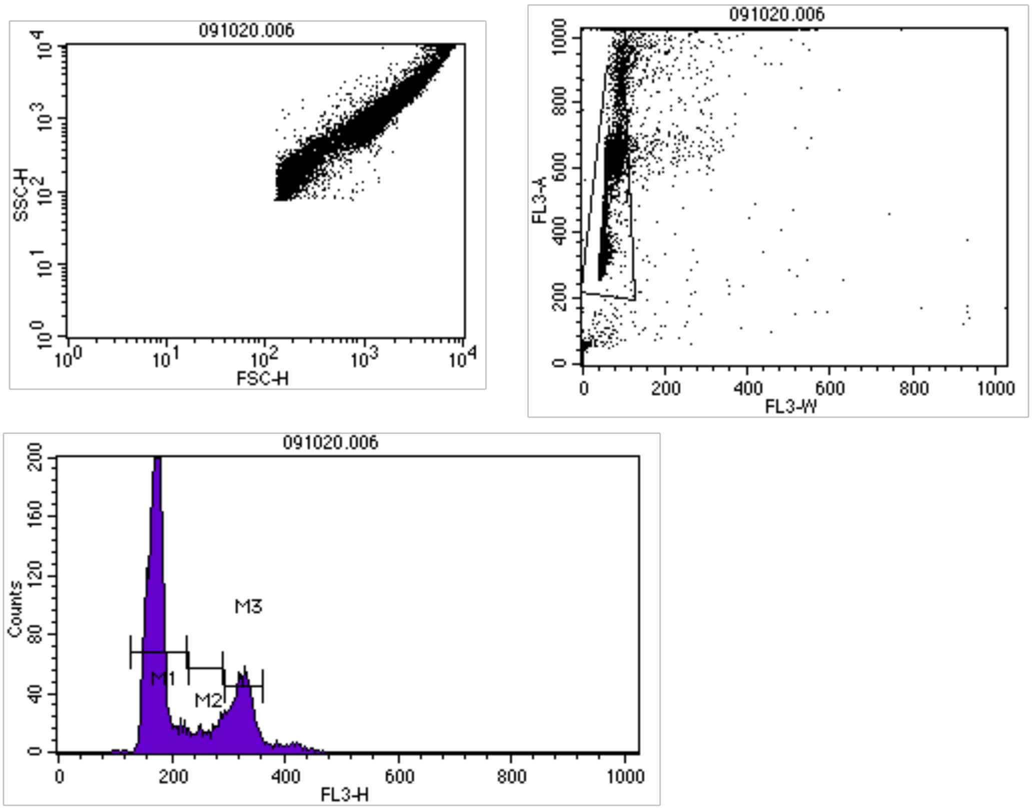

Marker	Left, Right	Events	% Gated	% Total	Mean	Geo Mean	CV	Median	Peak Ch
All	0, 1023	10000	100.00	64.32	214.44	205.17	32.04	178.00	174
M1	127, 227	7032	70.32	45.23	172.93	172.38	8.10	173.00	174
M2	230, 290	730	7.30	4.70	264.21	263.58	6.84	266.00	286
M3	294, 361	1900	19.00	12.22	323.89	323.51	4.81	325.00	328

Supplementary Figure 5b - In vivo 48h – G1 = 68.77

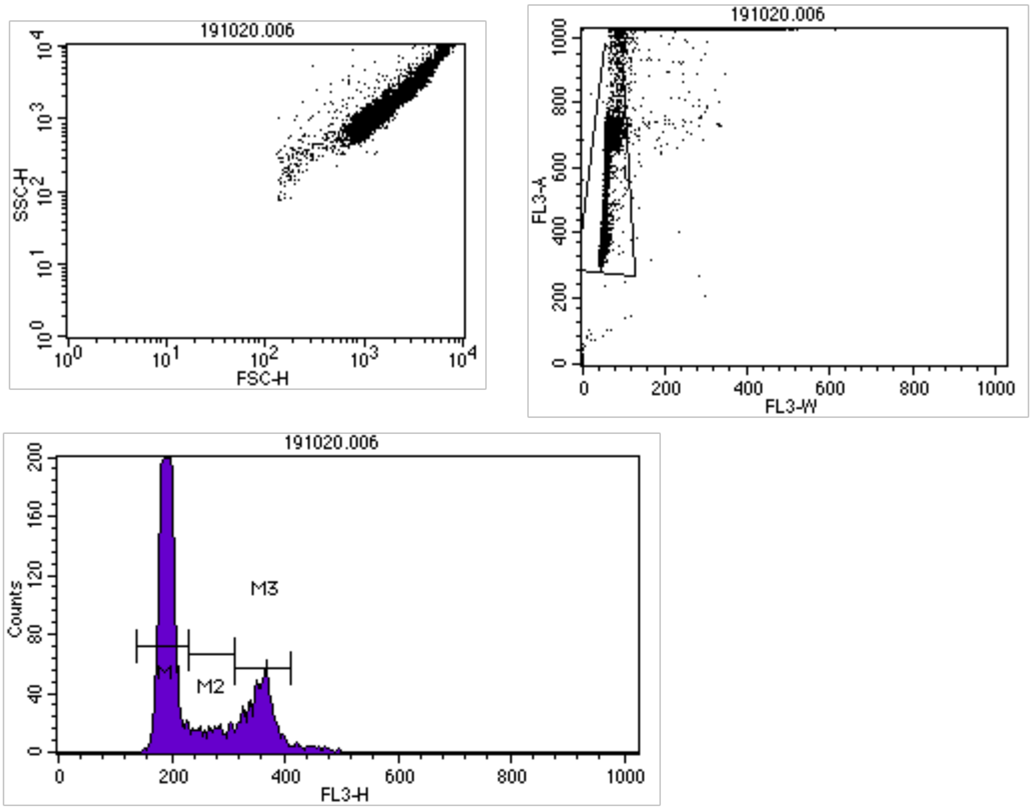

Marker	Left, Right	Events	% Gated	% Total	Mean	Geo Mean	CV	Median	Peak Ch
All	0, 1023	10000	100.00	83.78	236.39	227.31	30.19	198.00	192
M1	138, 230	6877	68.77	57.62	192.22	191.90	5.83	192.00	192
M2	230, 312	911	9.11	7.63	270.55	269.45	9.01	271.00	304
M3	312, 411	2130	21.30	17.85	354.66	354.08	5.69	356.00	365

Supplementary Figure 5b - In vivo 48h – G1 = 74.33

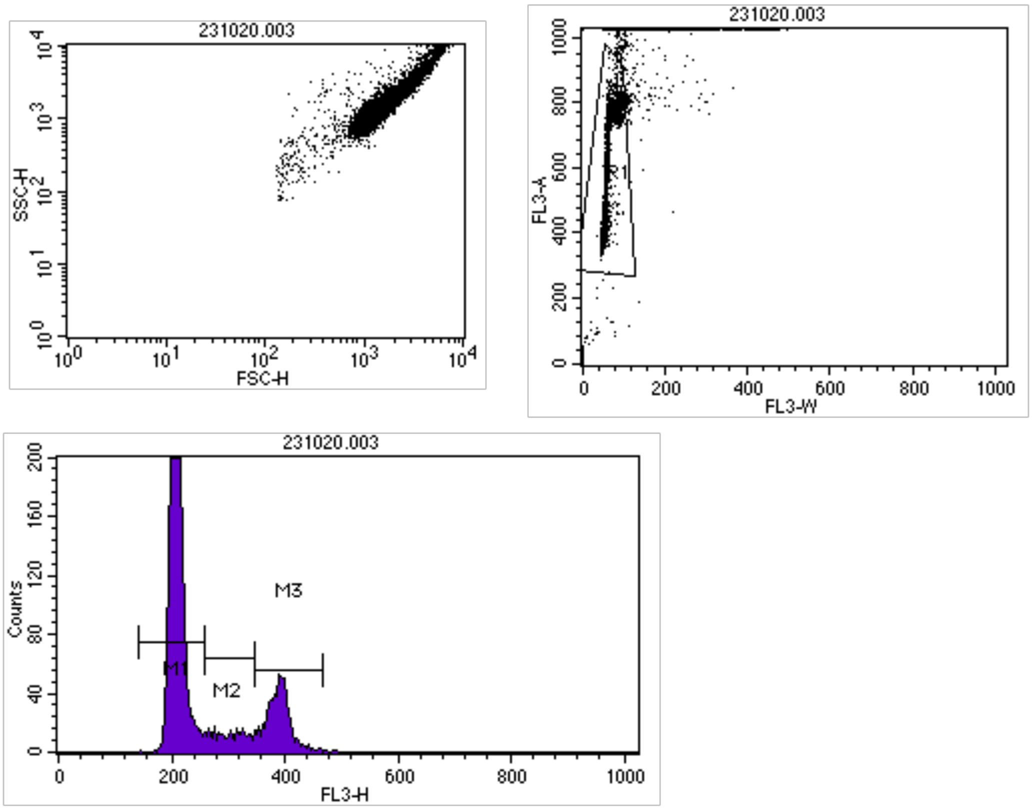

Marker	Left, Right	Events	% Gated	% Total	Mean	Geo Mean	CV	Median	Peak Ch
All	0, 1023	10000	100.00	85.91	249.08	240.65	28.68	212.00	209
M1	142, 258	7433	74.33	63.86	209.51	209.22	5.38	208.00	209
M2	258, 347	727	7.27	6.25	303.45	302.31	8.62	305.00	275
M3	347, 467	1848	18.48	15.88	386.71	386.27	4.79	387.00	388

Supplementary Figure 5b - In vivo 48h – G1 = 69.08

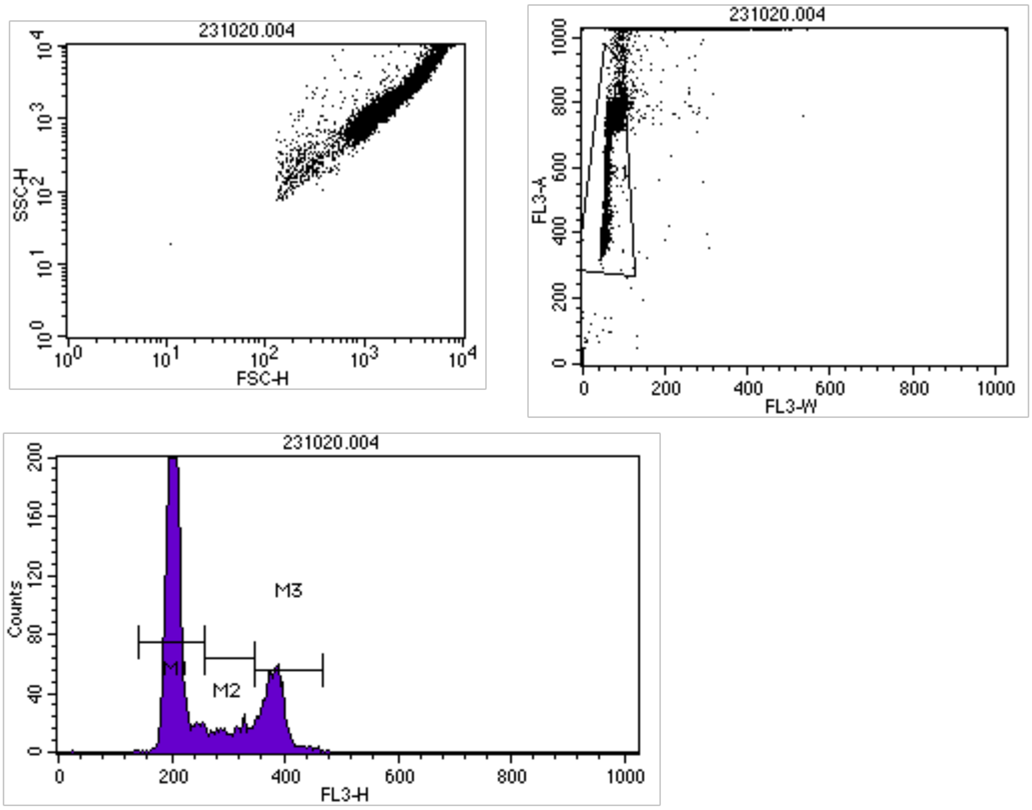

Marker	Left, Right	Events	% Gated	% Total	Mean	Geo Mean	CV	Median	Peak Ch
All	0, 1023	10000	100.00	80.04	251.72	242.33	29.42	209.00	206
M1	142, 258	6908	69.08	55.29	205.31	204.89	6.49	204.00	206
M2	258, 347	1014	10.14	8.12	304.75	303.58	8.70	307.00	327
M3	347, 467	2093	20.93	16.75	379.71	379.29	4.79	379.00	386

Supplementary Figure 5b - In vivo 48h – G1 = 71.01

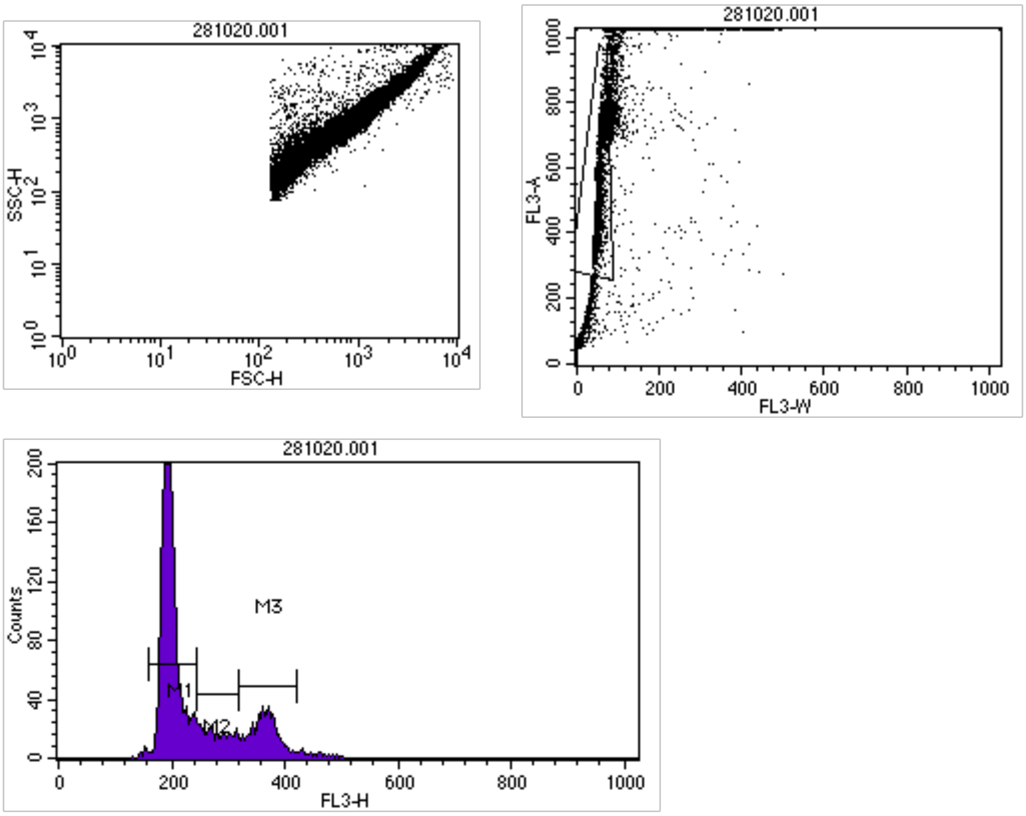

Marker	Left, Right	Events	% Gated	% Total	Mean	Geo Mean	CV	Median	Peak Ch
All	0, 1023	8699	100.00	55.82	236.08	227.93	28.89	201.00	193
M1	159, 244	6177	71.01	39.63	197.60	197.09	7.32	195.00	193
M2	244, 319	1007	11.58	6.46	278.23	277.33	8.06	276.00	247
M3	319, 421	1425	16.38	9.14	362.89	362.20	6.15	364.00	361

Supplementary Figure 5b - In vivo 48h – G1 = 71.64

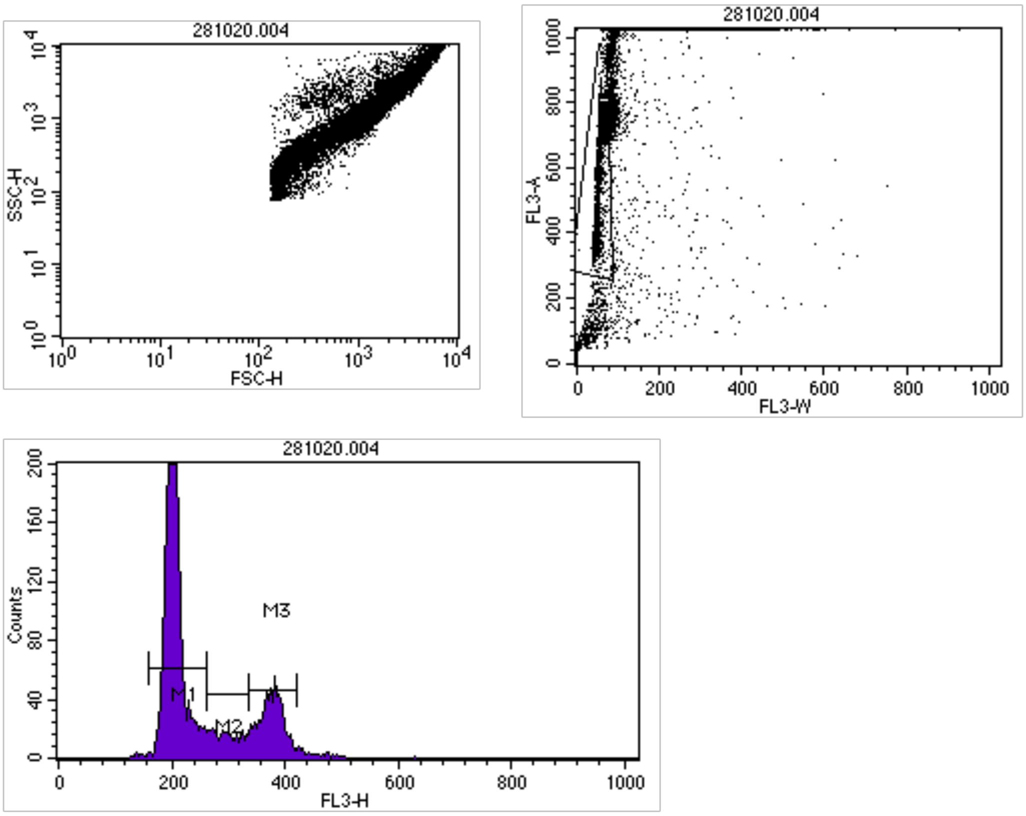

Marker	Left, Right	Events	% Gated	% Total	Mean	Geo Mean	CV	Median	Peak Ch
All	0, 1023	10000	100.00	56.61	246.75	237.76	29.42	208.00	201
M1	159, 262	7164	71.64	40.55	204.90	204.29	7.93	202.00	201
M2	262, 336	876	8.76	4.96	297.53	296.67	7.61	296.00	271
M3	336, 421	1865	18.65	10.56	374.26	373.79	5.04	375.00	379

Supplementary Figure 5b - In vivo 48h – G1 = 78.24

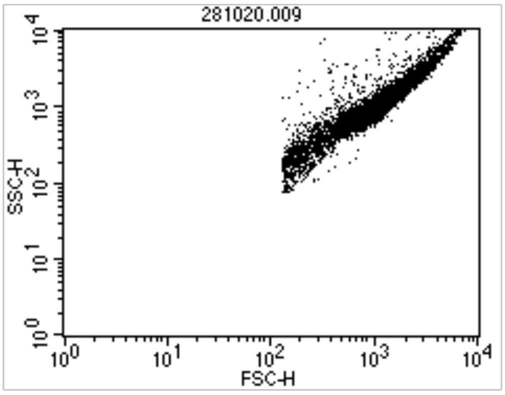

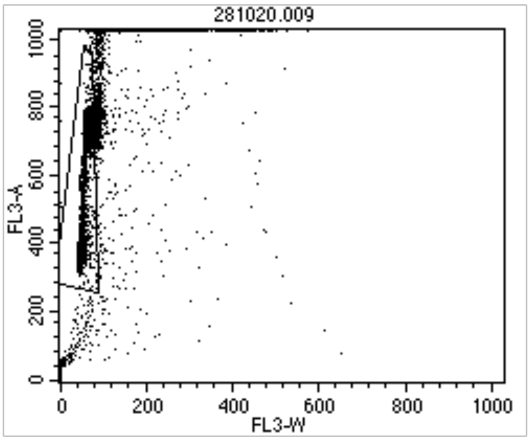

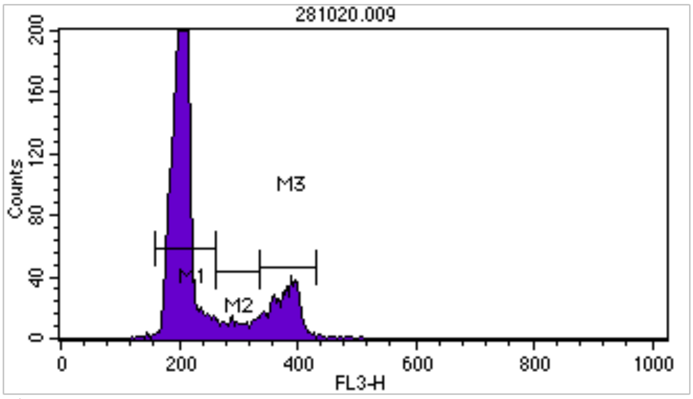

Marker	Left, Right	Events	% Gated	% Total	Mean	Geo Mean	CV	Median	Peak Ch
All	0, 1023	10000	100.00	77.96	237.44	229.67	28.51	208.00	209
M1	159, 262	7824	78.24	61.00	204.17	203.68	7.05	204.00	209
M2	262, 336	560	5.60	4.37	300.04	299.20	7.50	299.50	288
M3	336, 432	1591	15.91	12.40	377.23	376.68	5.40	380.00	388

Supplementary Figure 5b - Explant 24h – G1 = 63.72

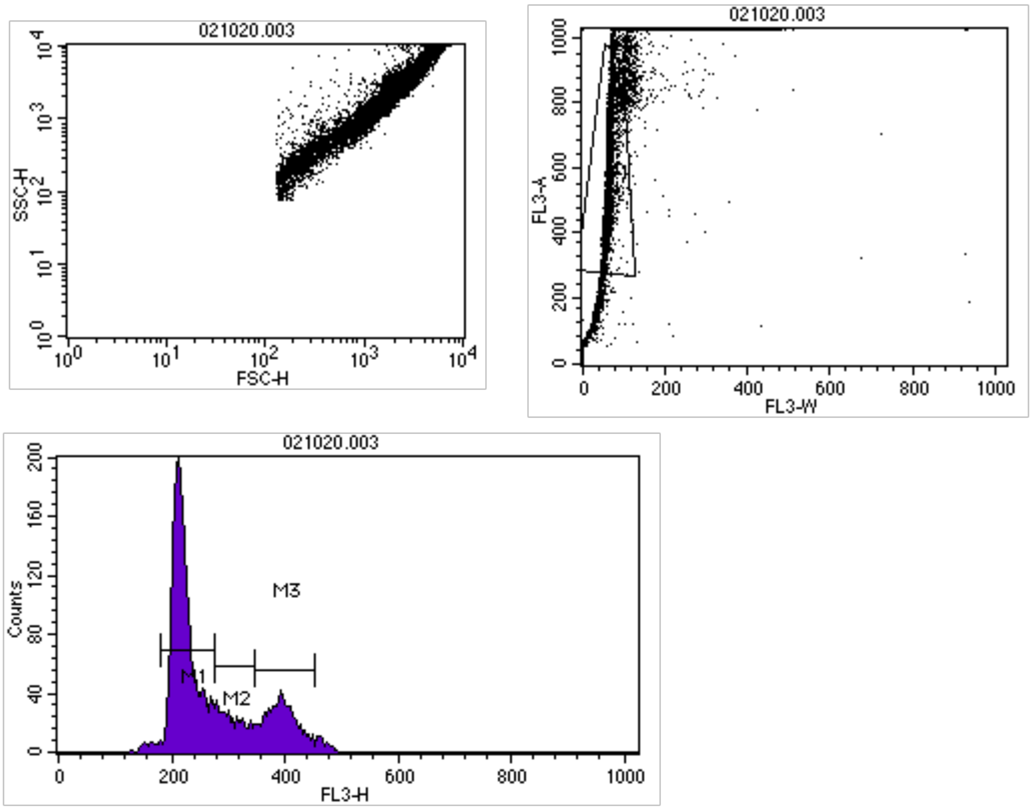

Marker	Left, Right	Events	% Gated	% Total	Mean	Geo Mean	CV	Median	Peak Ch
All	0, 1023	10000	100.00	64.61	270.85	261.09	28.52	232.00	213
M1	181, 276	6372	63.72	41.17	221.57	220.77	8.75	217.00	213
M2	276, 347	1303	13.03	8.42	307.55	306.86	6.75	305.00	278
M3	347, 453	2079	20.79	13.43	393.65	392.86	6.37	393.00	392

Supplementary Figure 5b - Explant 24h – G1 = 61.16

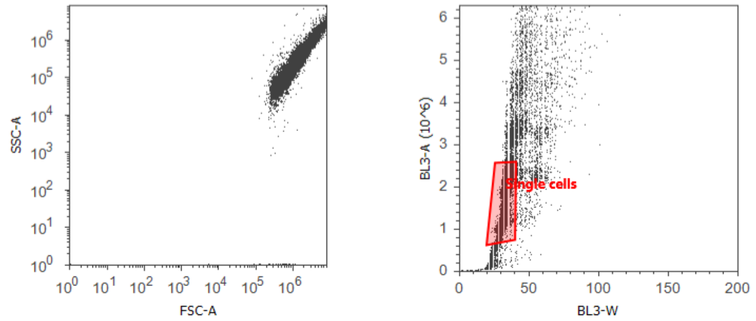

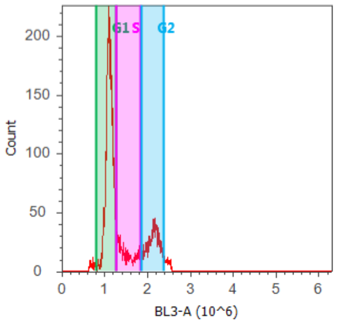

Gate: **Ungated**
Experiment Name: **061219 PI cell cycle(1)**
Sample Name: **hh20 posterior +24hr**

Name	Event Count	% Parent	% Total
All Events	16,714	100.000%	100.000%
Single cells	10,000	59.830%	59.830%
G1	6,116	61.160%	36.592%
S	1,370	13.700%	8.197%
G2	2,069	20.690%	12.379%

Supplementary Figure 5b - Explant 24h – G1 = 70.99 +FBS

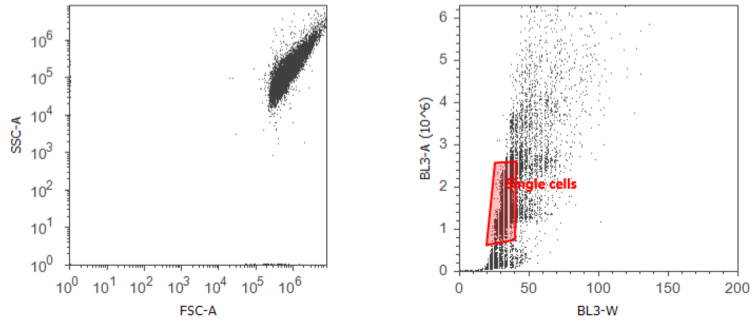

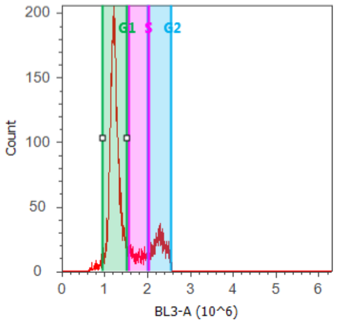

Gate: **Ungated**
Experiment Name: **201219 PI cell cycle**
Sample Name: **Posterior + FBS 24hr**

Name	Event Count	% Parent	% Total
All Events	17,663	100.000%	100.000%
Single cells	10,000	56.616%	56.616%
G1	7,099	70.990%	40.191%
S	943	9.430%	5.339%
G2	1,673	16.730%	9.472%

Supplementary Figure 5b - Explant 24h – G1 = 69.71

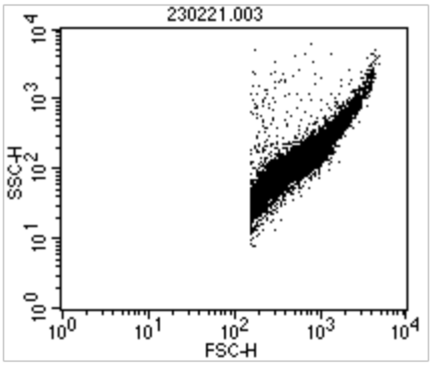

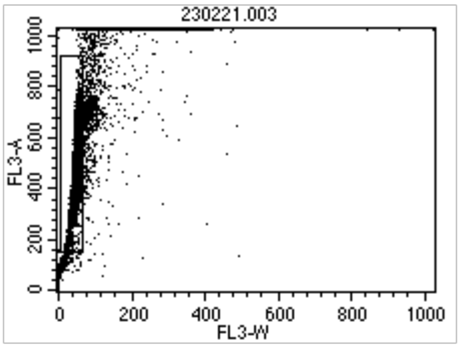

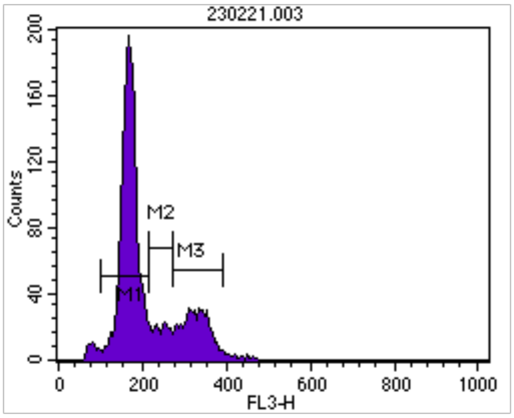

Marker	% Gated	% Total
All	100.00	71.48
M1	69.71	49.83
M2	8.03	5.74
M3	20.06	14.34

Supplementary Figure 5b - Explant 48h – G1 = 71.19

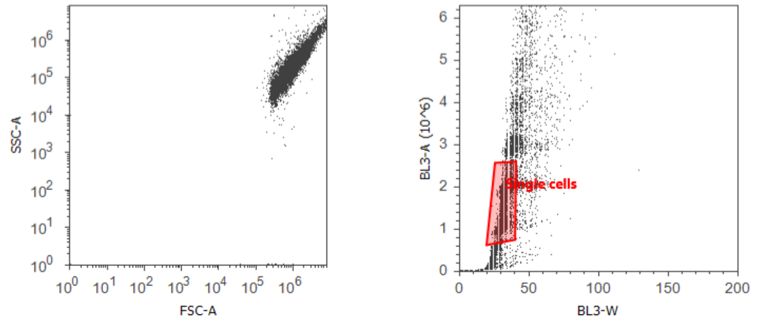

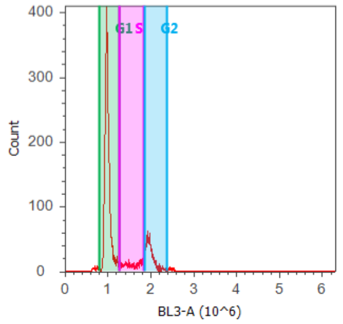

Gate: **Ungated**
Experiment Name: **061219 PI cell cycle(1)**
Sample Name: **hh20 posterior explant 48hr**

Name	Event Count	% Parent	% Total
All Events	15,382	100.000%	100.000%
Single cells	10,000	65.011%	65.011%
G1	7,119	71.190%	46.281%
S	962	9.620%	6.254%
G2	1,611	16.110%	10.473%

Supplementary Figure 5b - Explant 48h – G1 = 76.77

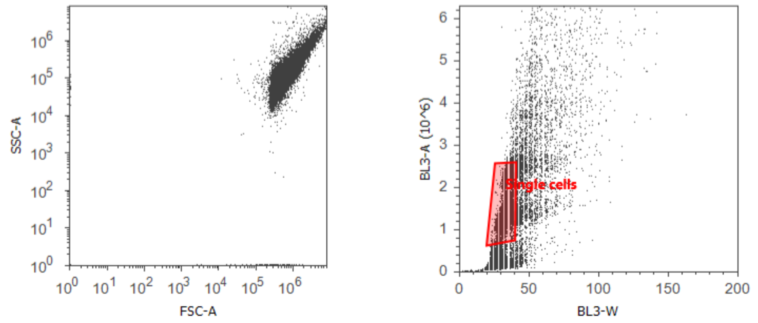

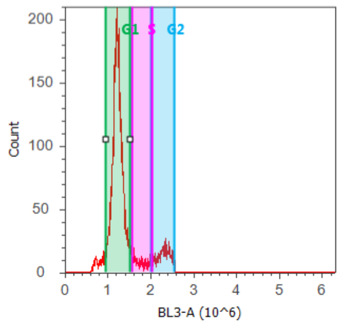

Gate: **Ungated**
Experiment Name: **201219 PI cell cycle**
Sample Name: **Posterior +FBS 48hr**

Name	Event Count	% Parent	% Total
All Events	25,013	100.000%	100.000%
Single cells	10,000	39.979%	39.979%
G1	7,677	76.770%	30.692%
S	610	6.100%	2.439%
G2	1,165	11.650%	4.658%

Supplementary Figure 5b - Explant 48h – G1 = 79.4

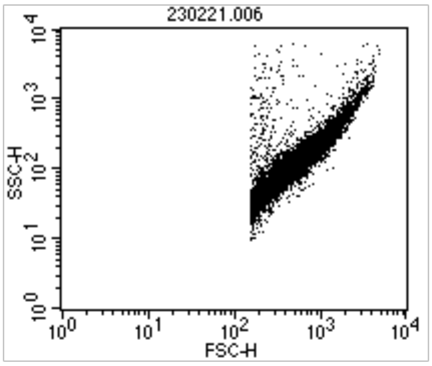

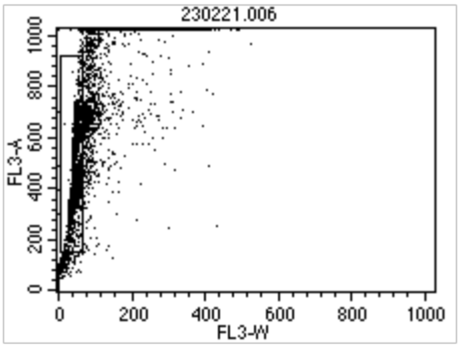

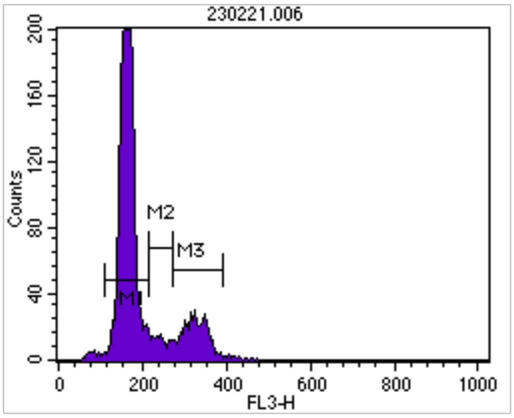

Marker	% Gated	% Total
All	100.00	71.19
M1	79.40	56.53
M2	5.22	3.72
M3	14.18	10.10

Figure 3m - DMSO 24h – G1 = 66.72

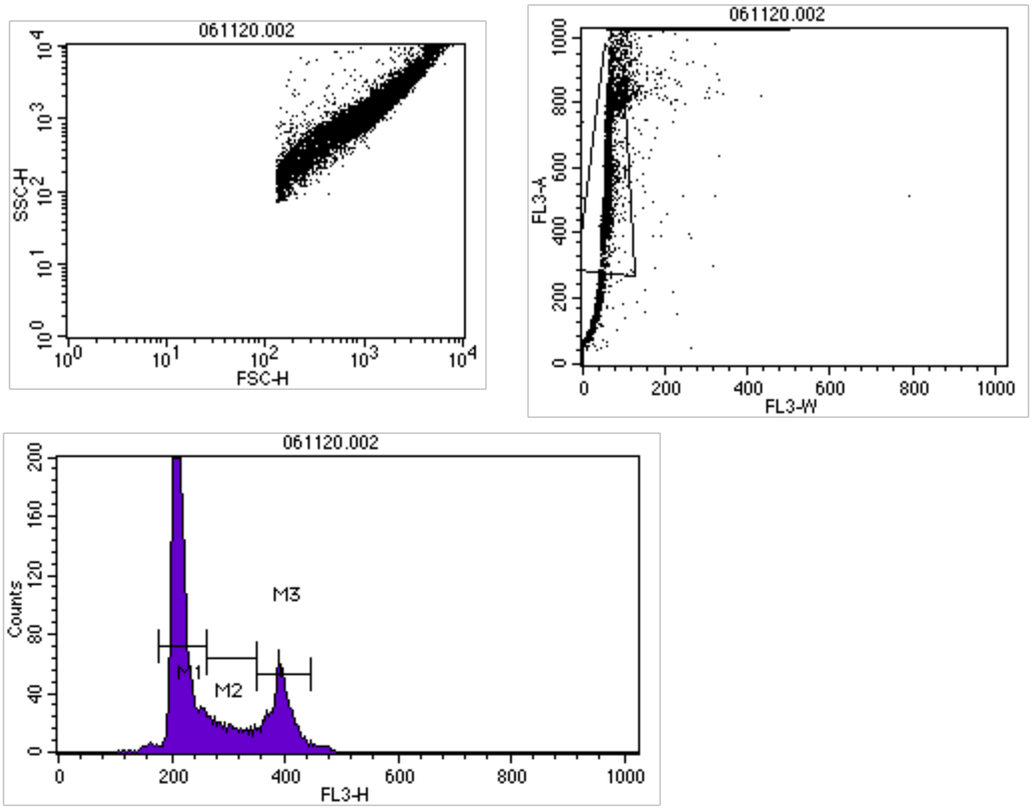

Marker	Left, Right	Events	% Gated	% Total	Mean	Geo Mean	CV	Median	Peak Ch
All	0, 1023	10000	100.00	67.33	262.63	253.15	28.90	220.00	210
M1	177, 262	6672	66.72	44.92	215.29	214.85	6.55	212.00	210
M2	262, 350	1145	11.45	7.71	303.65	302.54	8.59	302.00	265
M3	350, 446	2059	20.59	13.86	391.85	391.33	5.14	392.00	389

Figure 3m - DMSO 24h – G1 = 69.69

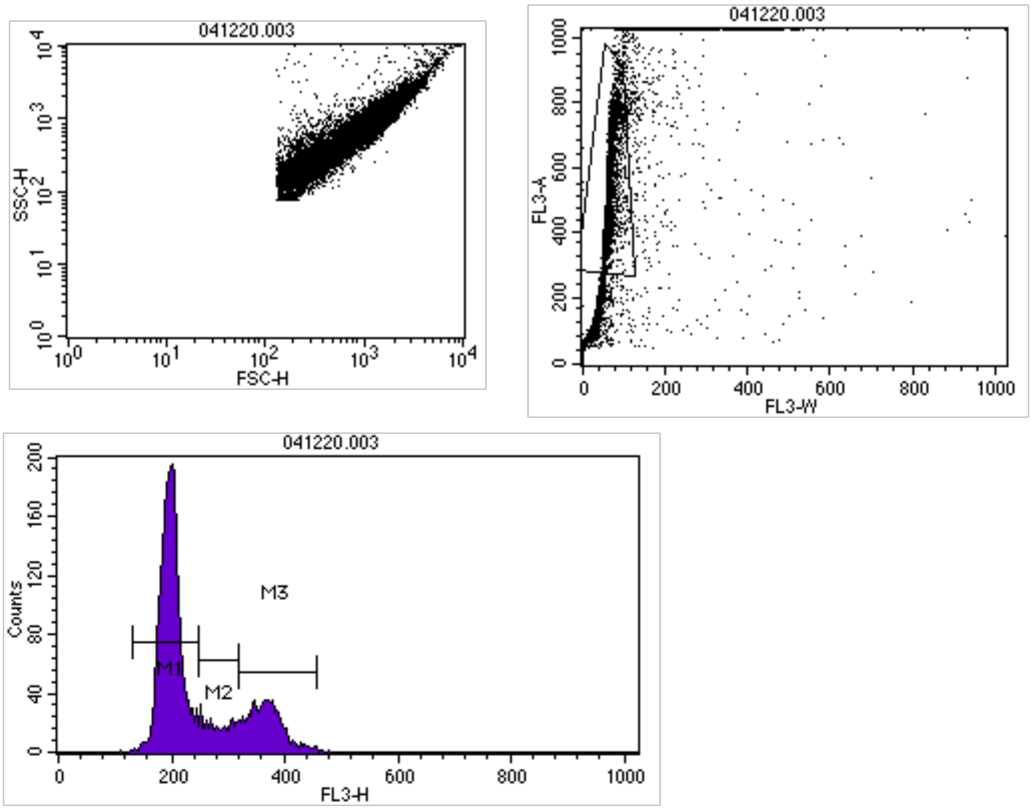

Marker	Left, Right	Events	% Gated	% Total	Mean	Geo Mean	CV	Median	Peak Ch
All	0, 1023	10000	100.00	63.10	240.38	231.55	29.30	205.00	203
M1	131, 248	6969	69.69	43.98	198.09	197.37	8.60	197.00	203
M2	248, 319	1026	10.26	6.47	283.67	282.82	7.72	284.00	252
M3	319, 457	2028	20.28	12.80	364.18	363.21	7.37	363.00	368

Figure 3m - DMSO 24h – G1 = 66.98

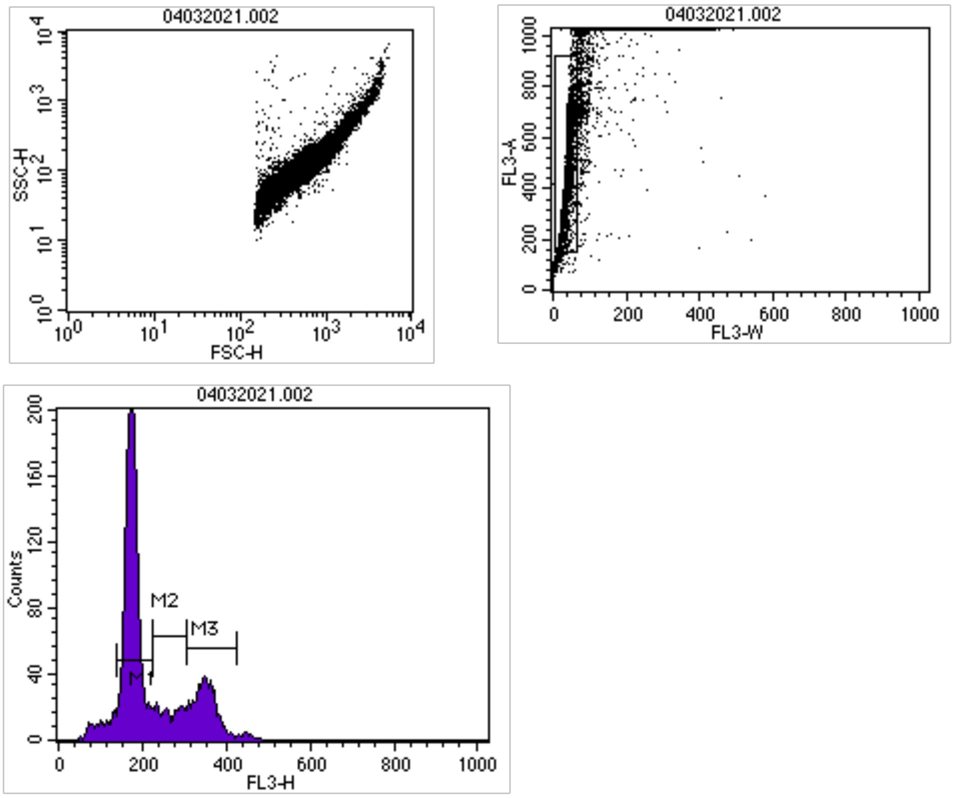

Marker	% Gated	% Total
All	100.00	76.30
M1	66.98	51.11
M2	9.90	7.55
M3	17.86	13.63

Figure 3m - DMSO 24h – G1 = 67.98

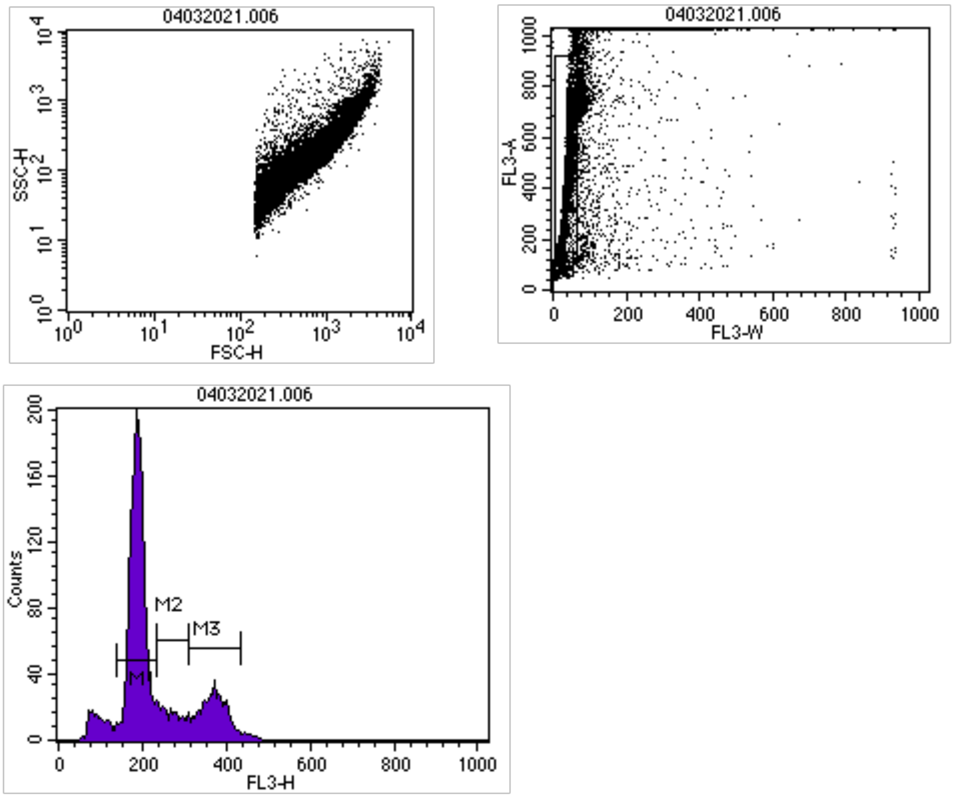

Marker	Events	% Gated	% Total
All	10000	100.00	45.33
M1	6798	67.98	30.82
M2	874	8.74	3.96
M3	1694	16.94	7.68

Figure 3m - DMSO 48h – G1 = 75.04

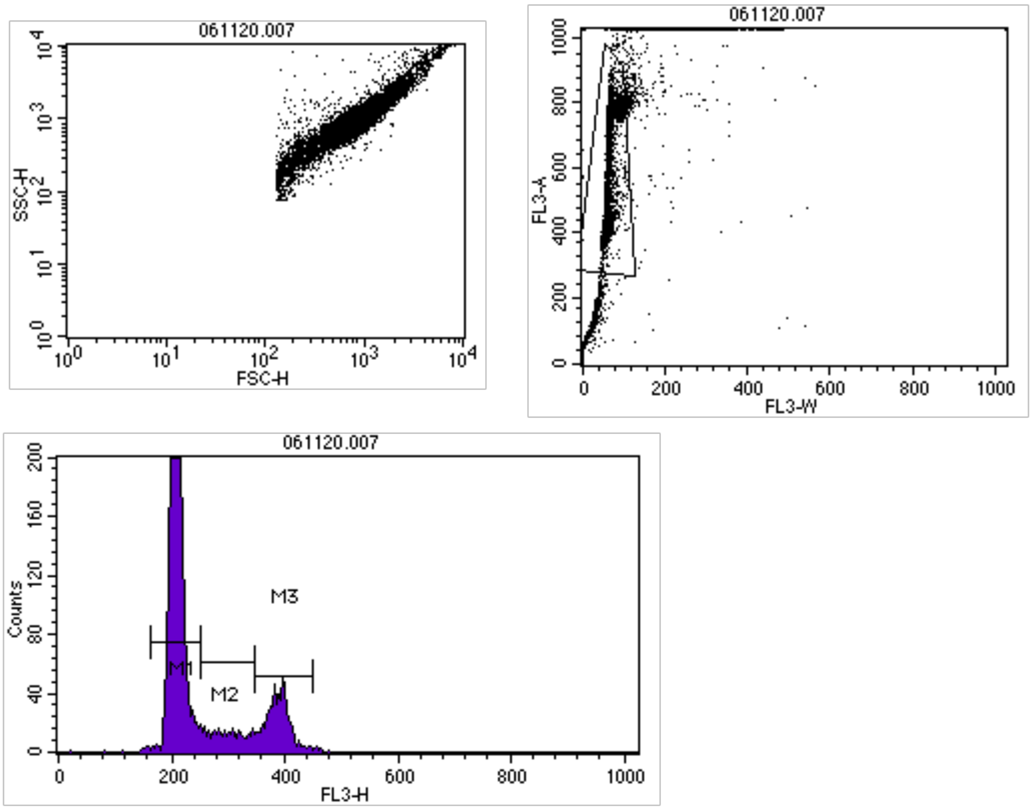

Marker	Left, Right	Events	% Gated	% Total	Mean	Geo Mean	CV	Median	Peak Ch
All	0, 1023	10000	100.00	77.72	244.57	236.72	28.04	211.00	207
M1	163, 251	7504	75.04	58.32	208.54	208.24	5.40	207.00	207
M2	251, 347	889	8.89	6.91	297.90	296.49	9.70	298.00	251
M3	347, 450	1590	15.90	12.36	385.67	385.22	4.82	386.00	394

Figure 3m - DMSO 48h – G1 = 76.58

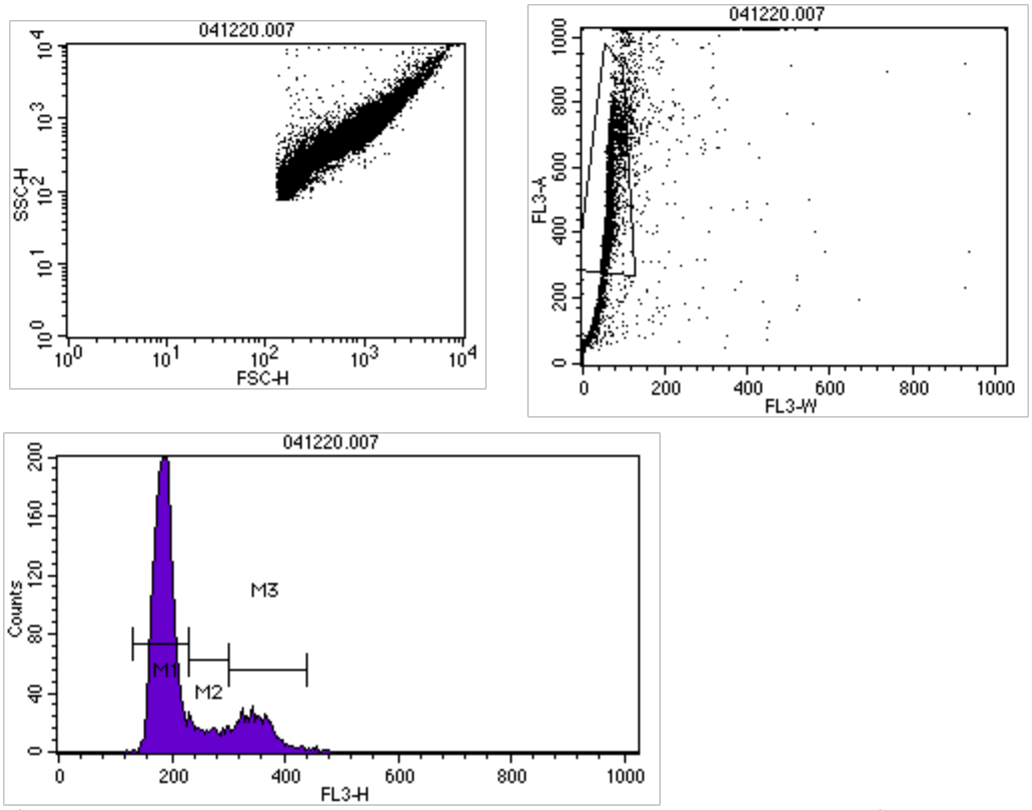

Marker	Left, Right	Events	% Gated	% Total	Mean	Geo Mean	CV	Median	Peak Ch
All	0, 1023	10000	100.00	65.55	217.35	210.06	28.90	192.00	191
M1	131, 230	7658	76.58	50.20	186.06	185.43	8.24	186.00	191
M2	230, 301	818	8.18	5.36	262.76	261.85	8.33	262.00	231
M3	301, 439	1538	15.38	10.08	346.96	345.88	7.98	344.00	341

Figure 3m - DMSO 48h – G1 = 74.63

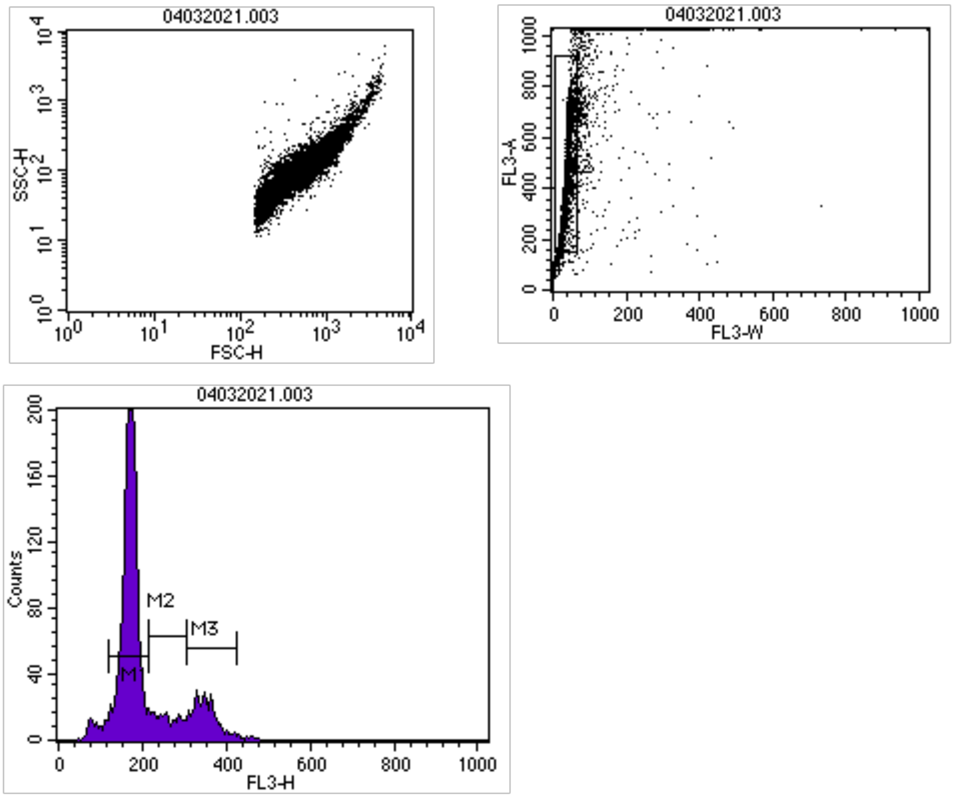

Marker	% Gated	% Total
All	100.00	78.54
M1	74.63	58.62
M2	9.15	7.19
M3	12.55	9.86

Figure 3m - DMSO 48h – G1 = 81.58

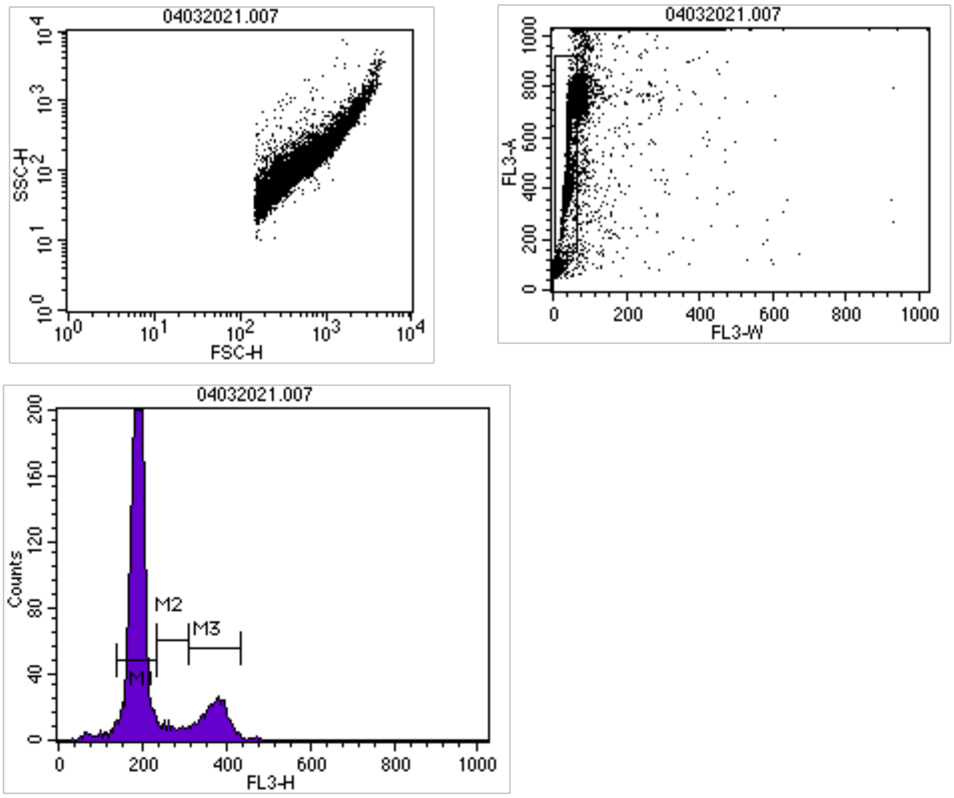

Marker	Events	% Gated	% Total
All	10000	100.00	64.65
M1	8158	81.58	52.74
M2	419	4.19	2.71
M3	1259	12.59	8.14

Figure 3m - SU5402 24h – G1 = 74.74

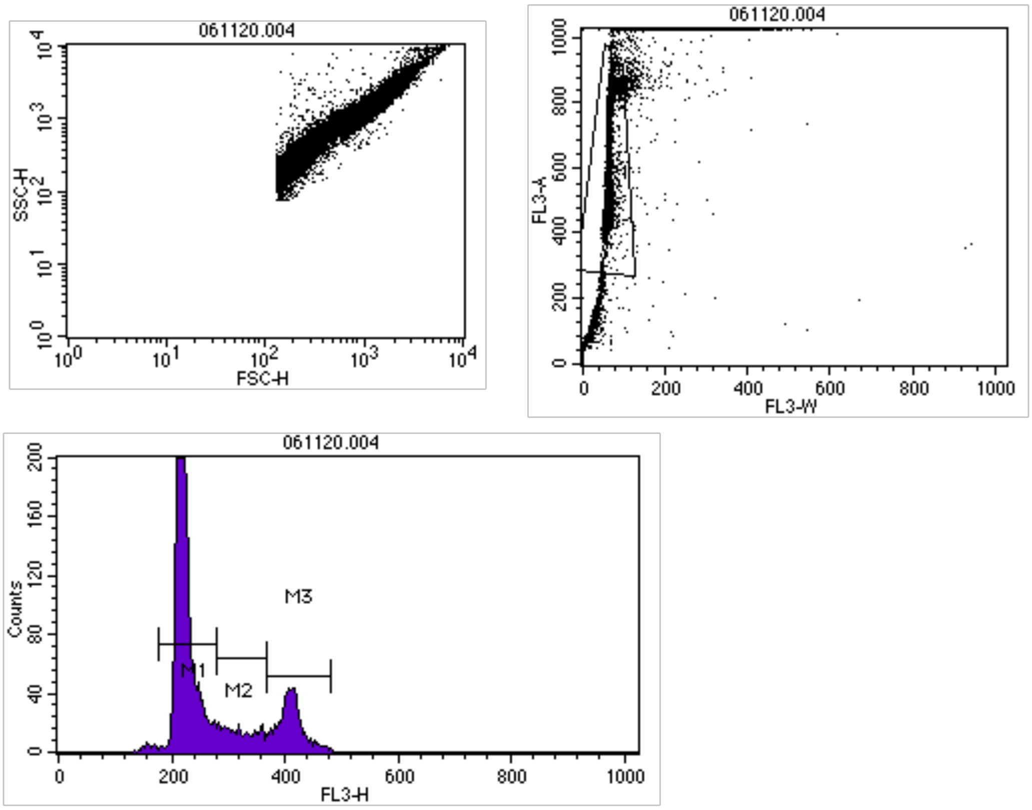

Marker	Left, Right	Events	% Gated	% Total	Mean	Geo Mean	CV	Median	Peak Ch
All	0, 1023	10000	100.00	58.91	260.24	252.00	27.70	223.00	218
M1	177, 280	7474	74.74	44.03	222.74	222.25	6.89	219.00	218
M2	280, 368	923	9.23	5.44	321.11	320.03	8.24	319.00	283
M3	368, 481	1562	15.62	9.20	408.70	408.22	4.89	408.00	413

Figure 3m - SU5402 24h – G1 = 69.47

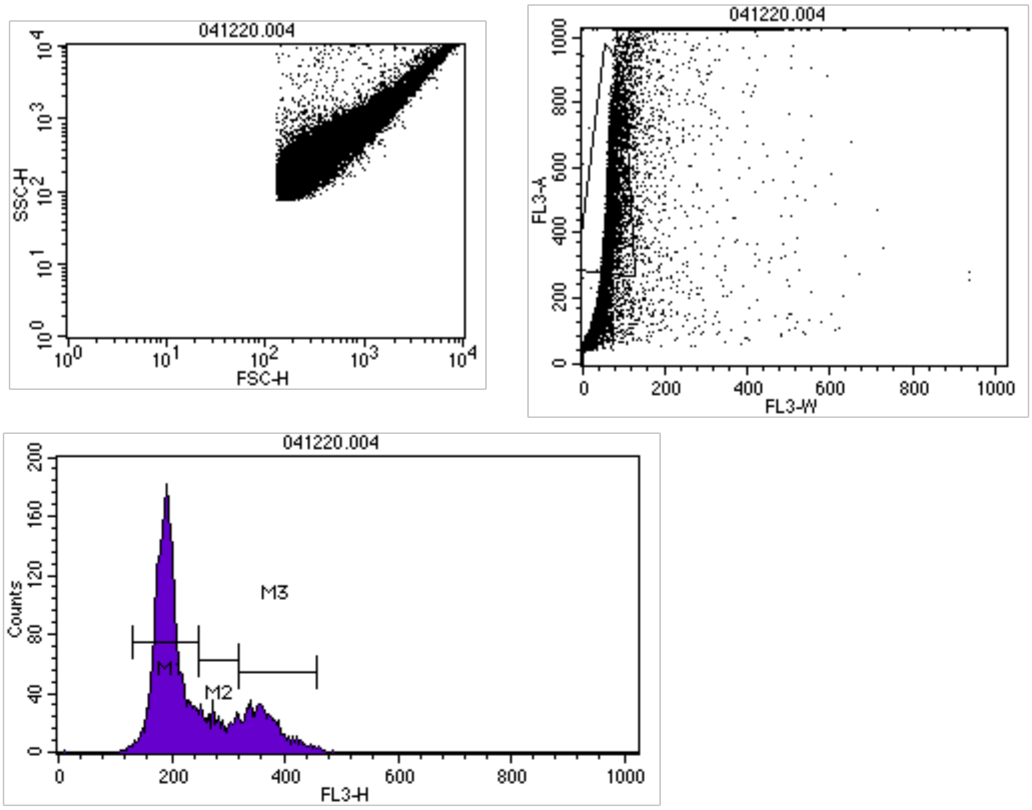

Marker	Left, Right	Events	% Gated	% Total	Mean	Geo Mean	CV	Median	Peak Ch
All	0, 1023	10000	100.00	34.63	234.37	225.06	30.37	201.00	192
M1	131, 248	6947	69.47	24.05	192.70	191.60	10.74	191.00	192
M2	248, 319	1269	12.69	4.39	281.63	280.80	7.70	279.00	271
M3	319, 457	1791	17.91	6.20	362.93	361.76	8.20	359.00	338

Figure 3m - SU5402 24h – G1 = 67.82

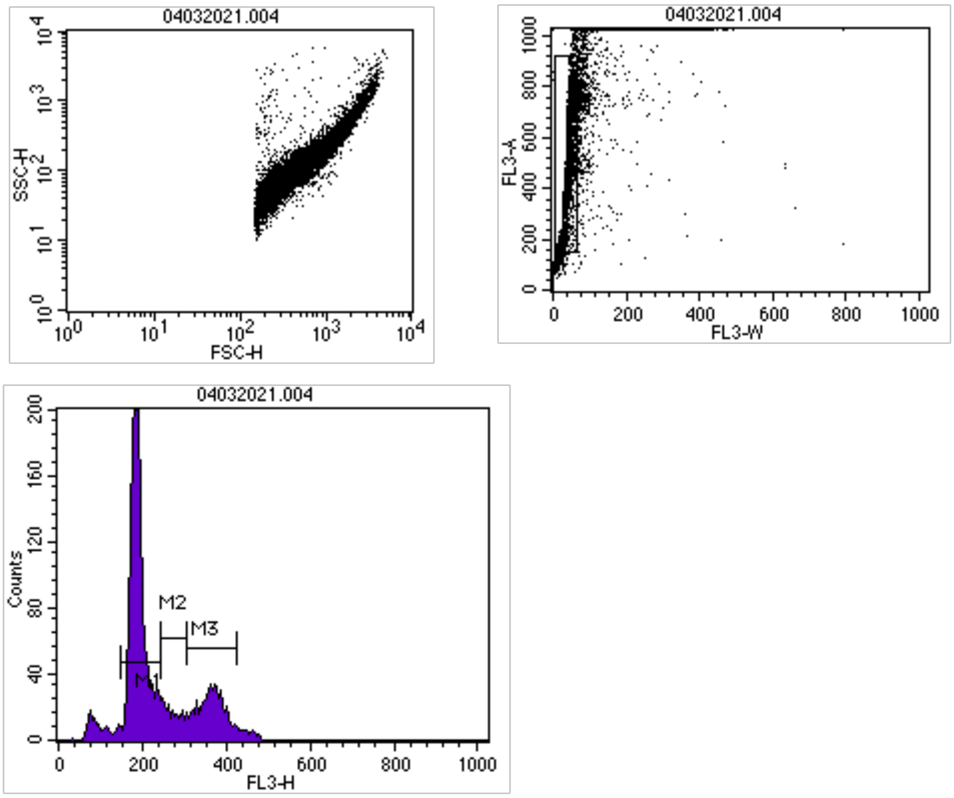

Marker	% Gated	% Total
All	100.00	66.03
M1	67.82	44.78
M2	7.85	5.18
M3	18.89	12.47

Figure 3m - SU5402 24h – G1 = 68.82

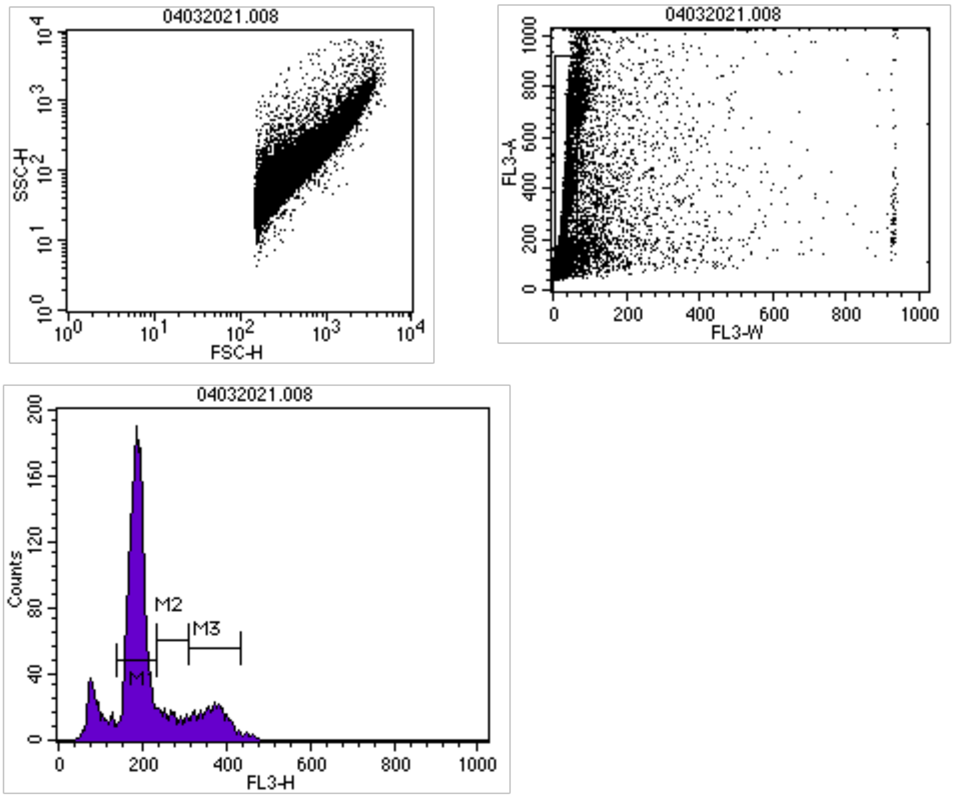

Marker	Events	% Gated	% Total
All	10000	100.00	31.44
M1	6882	68.82	21.64
M2	771	7.71	2.42
M3	1293	12.93	4.06

Figure 3m - SU5402 48h – G1 = 79.32

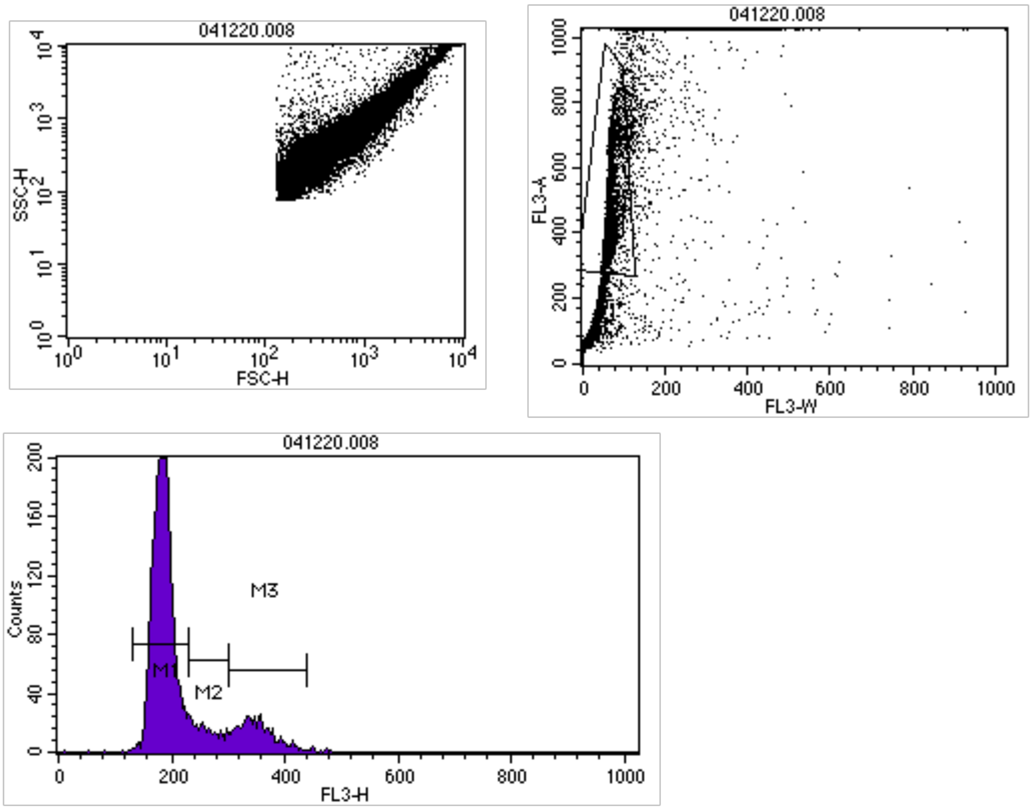

Marker	Left, Right	Events	% Gated	% Total	Mean	Geo Mean	CV	Median	Peak Ch
All	0, 1023	10000	100.00	37.02	211.34	204.80	27.89	189.00	186
M1	131, 230	7932	79.32	29.37	184.68	183.99	8.66	184.00	186
M2	230, 301	820	8.20	3.04	260.87	260.02	8.14	258.00	233
M3	301, 439	1259	12.59	4.66	346.09	344.99	8.10	343.00	356

Figure 3m - SU5402 48h – G1 = 77.53

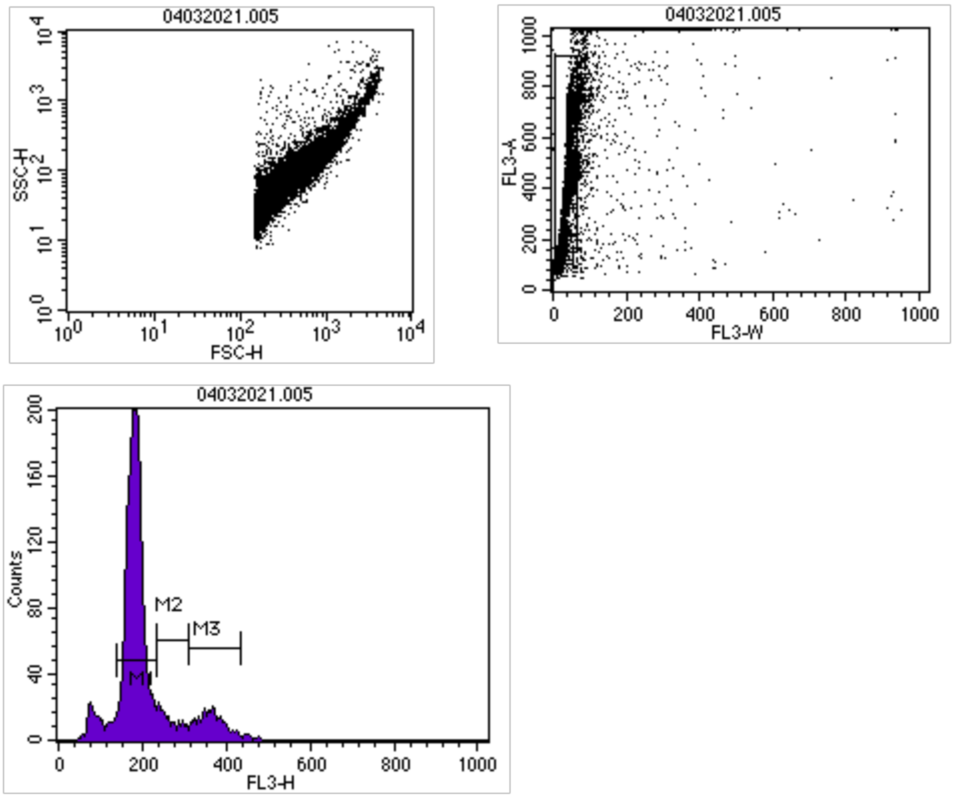

Marker	Events	% Gated	% Total
All	10000	100.00	48.37
M1	7753	77.53	37.50
M2	646	6.46	3.12
M3	922	9.22	4.46

Figure 3m - SU5402 48h – G1 = 84.05

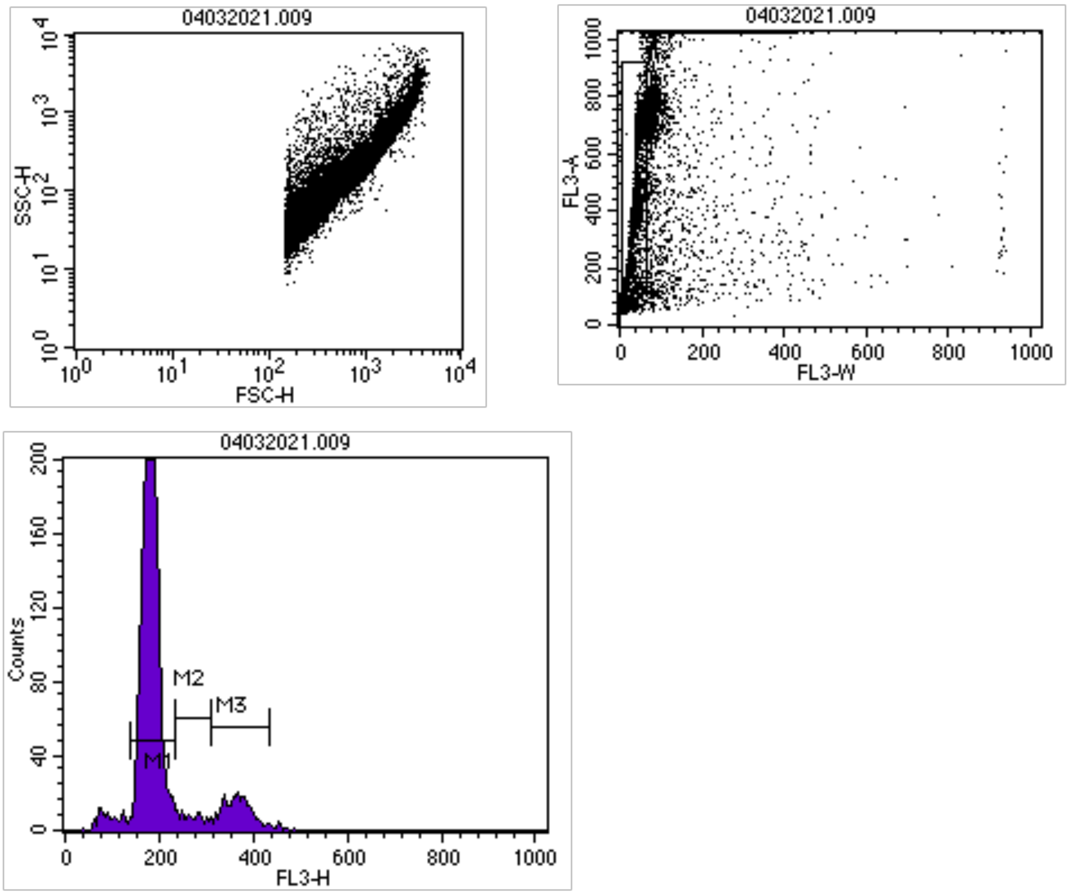

Marker	Events	% Gated	% Total
All	10000	100.00	33.77
M1	8405	84.05	28.39
M2	348	3.48	1.18
M3	923	9.23	3.12

Figure 3m - SU5402 48h – G1 = 84.30

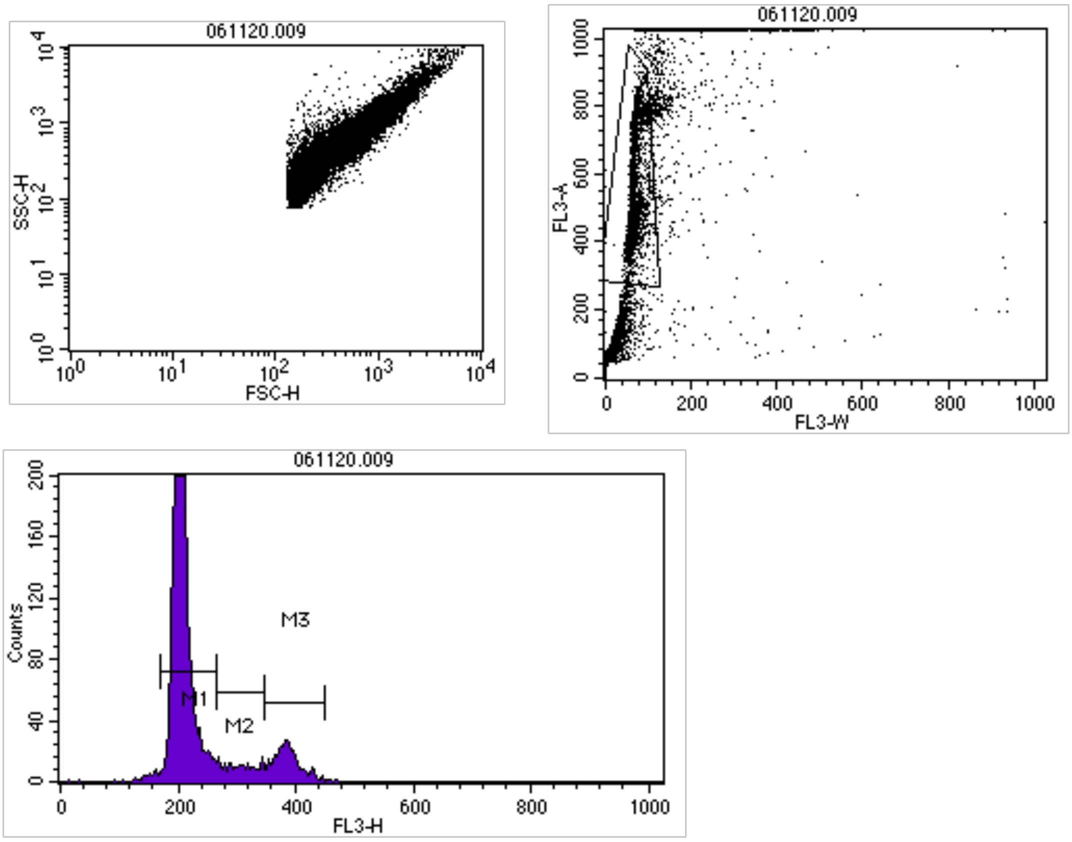

Marker	Left, Right	Events	% Gated	% Total	Mean	Geo Mean	CV	Median	Peak Ch
All	0, 1023	10000	100.00	44.82	227.49	221.81	25.41	205.00	200
M1	170, 265	8430	84.30	37.78	206.02	205.56	6.84	203.00	200
M2	265, 347	524	5.24	2.35	303.62	302.63	8.07	303.00	267
M3	347, 450	945	9.45	4.24	384.00	383.50	5.14	382.00	381

Figure 3a - +AER 24h – G1 = 69.57

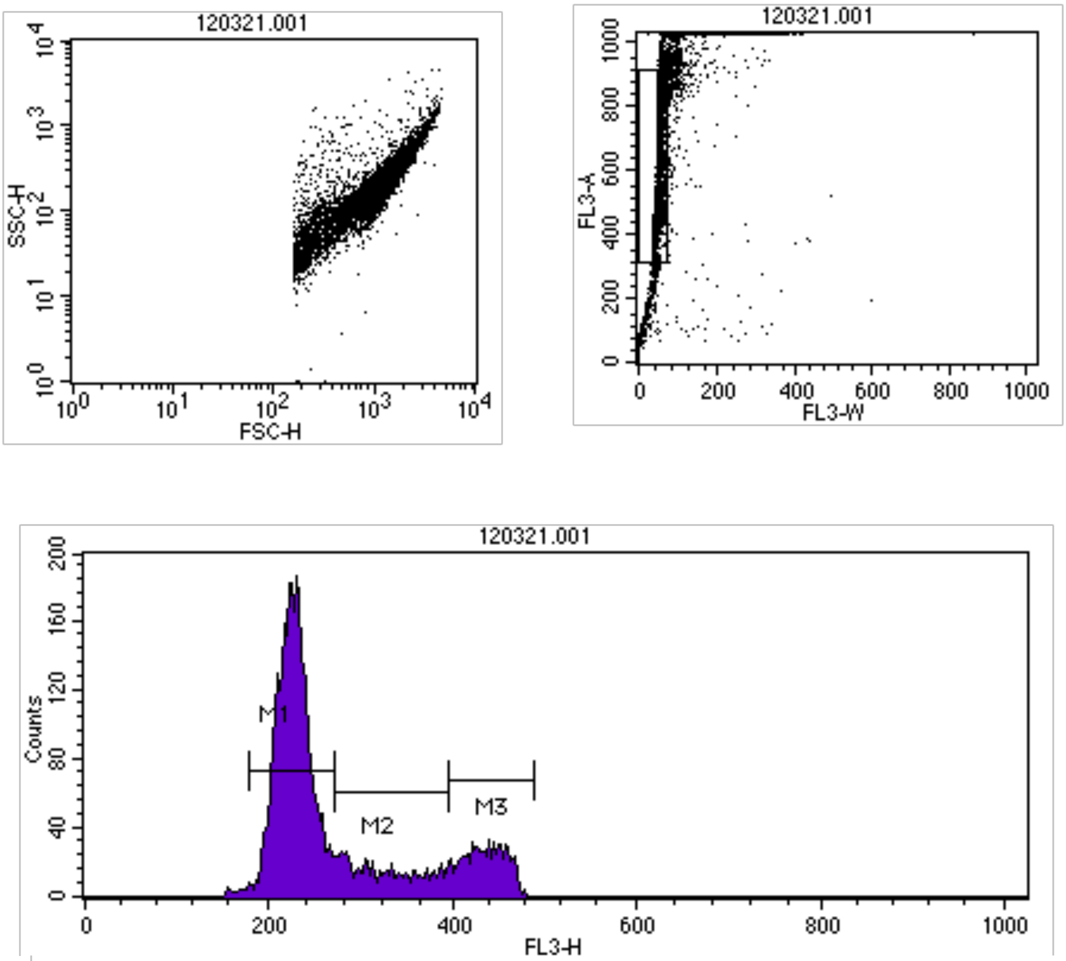

Marker	Left, Right	Events	% Gated	% Total	Mean	Geo Mean	CV	Median	Peak Ch
All	0, 1023	10000	100.00	73.45	272.73	262.87	29.35	235.00	229
M1	178, 271	6946	69.46	51.02	226.58	225.94	7.53	226.00	229
M2	271, 395	1504	15.04	11.05	328.05	325.82	11.69	325.00	284
M3	395, 488	1534	15.34	11.27	433.05	432.58	4.68	434.00	438

Figure 3a - +AER 24h – G1 = 69.4

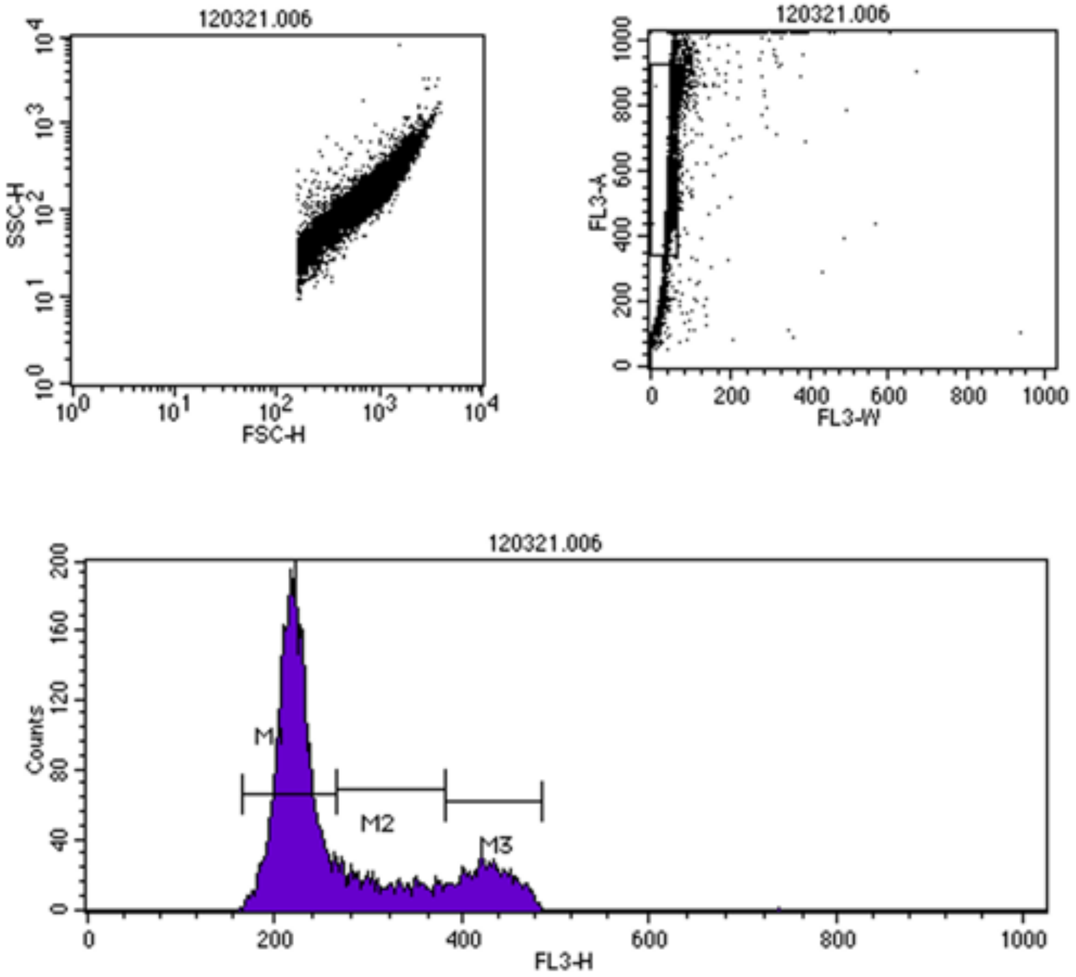

Marker	Left, Right	Events	% Gated	% Total	Mean	Geo Mean	CV	Median	Peak Ch
All	0, 1023	10000	100.00	69.02	267.97	257.88	30.05	229.00	221
M1	165, 266	6966	69.66	48.08	220.82	220.13	7.89	220.00	221
M2	266, 382	1484	14.84	10.24	319.31	317.36	11.09	318.00	270
M3	382, 486	1588	15.88	10.96	427.61	426.94	5.61	428.00	421

Figure 3a - +AER 24h – G1 = 68.89

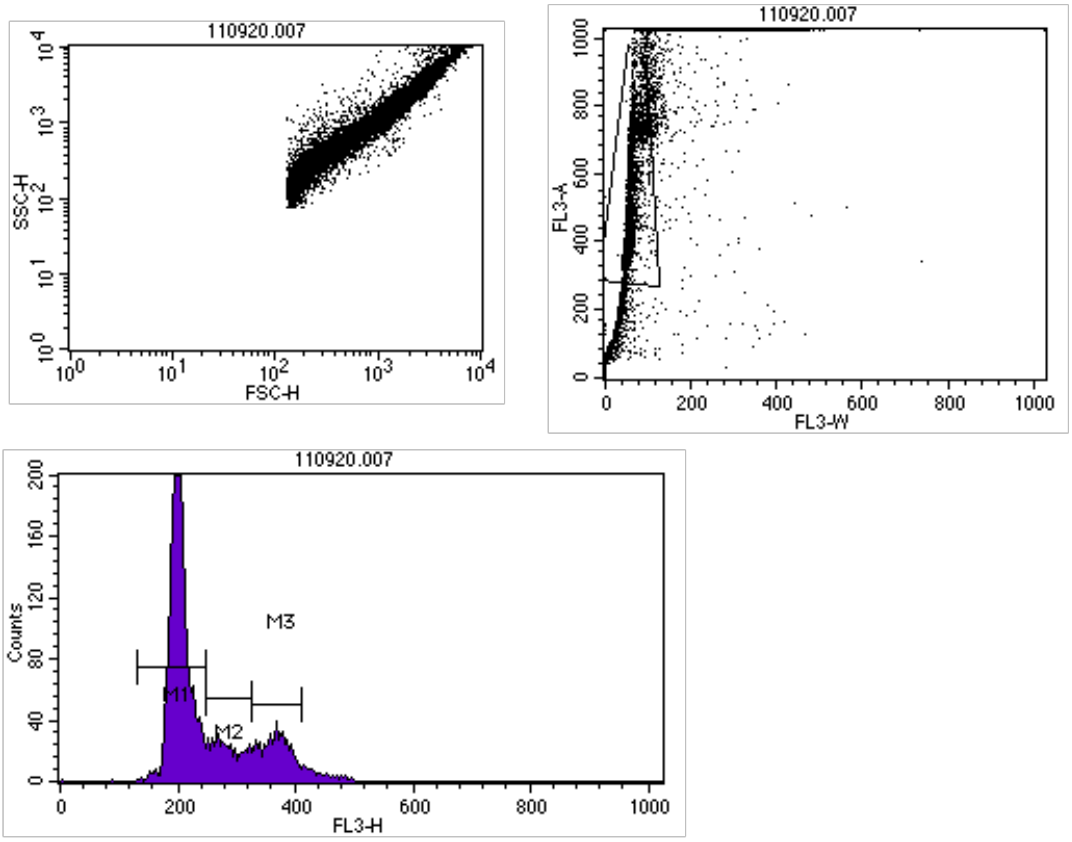

Marker	Left, Right	Events	% Gated	% Total	Mean	Geo Mean	CV	Median	Peak Ch
All	0, 1023	10000	100.00	57.34	244.22	235.87	28.42	209.00	198
M1	131, 248	6889	68.89	39.50	203.09	202.50	7.62	201.00	198
M2	248, 326	1369	13.69	7.85	283.23	282.33	7.98	281.00	269
M3	326, 411	1548	15.48	8.88	365.89	365.27	5.80	366.00	366

Figure 3a - +AER 48h – G1 = 81.05

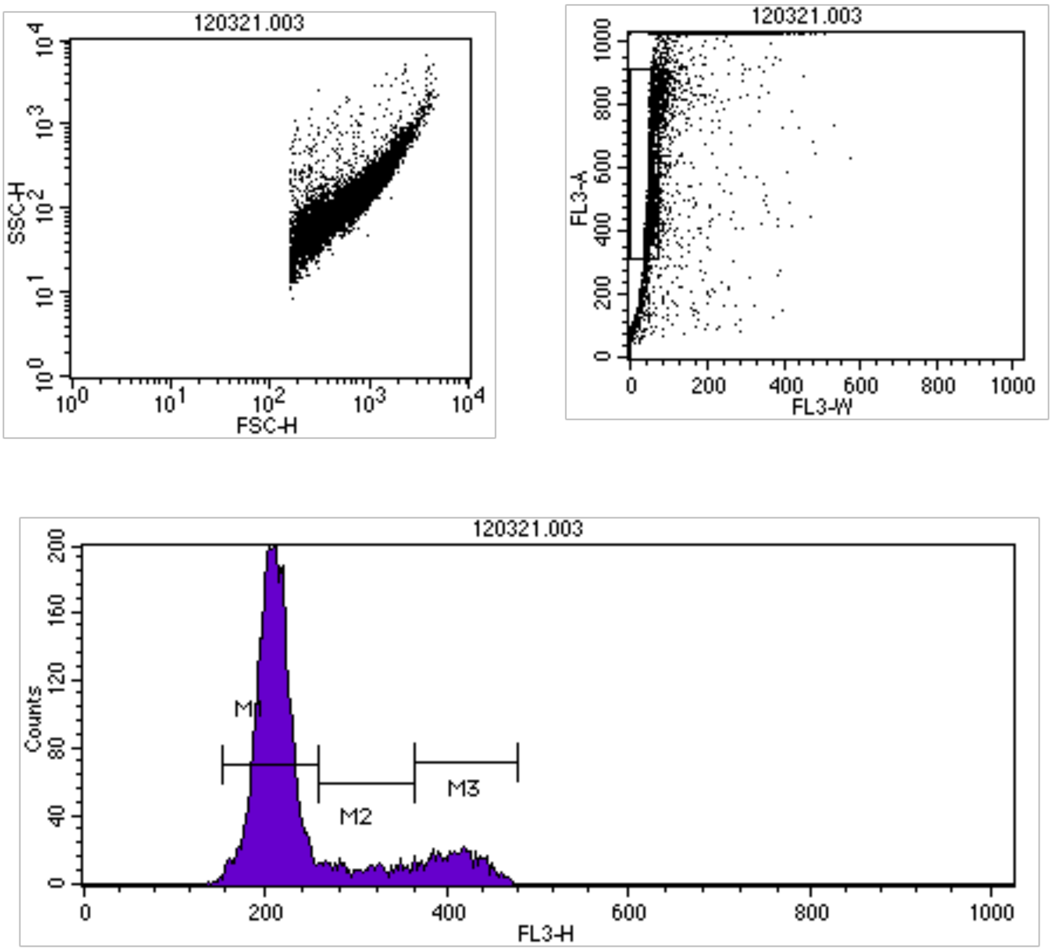

Marker	Left, Right	Events	% Gated	% Total	Mean	Geo Mean	CV	Median	Peak Ch
All	0, 1023	10000	100.00	73.02	238.71	230.91	29.06	213.00	204
M1	152, 258	8116	81.16	59.27	208.67	207.92	8.42	209.00	204
M2	258, 364	782	7.82	5.71	307.90	306.14	10.72	307.50	363
M3	364, 478	1116	11.16	8.15	410.84	410.06	6.19	410.50	417

Figure 3a - +AER 48h – G1 = 76.53

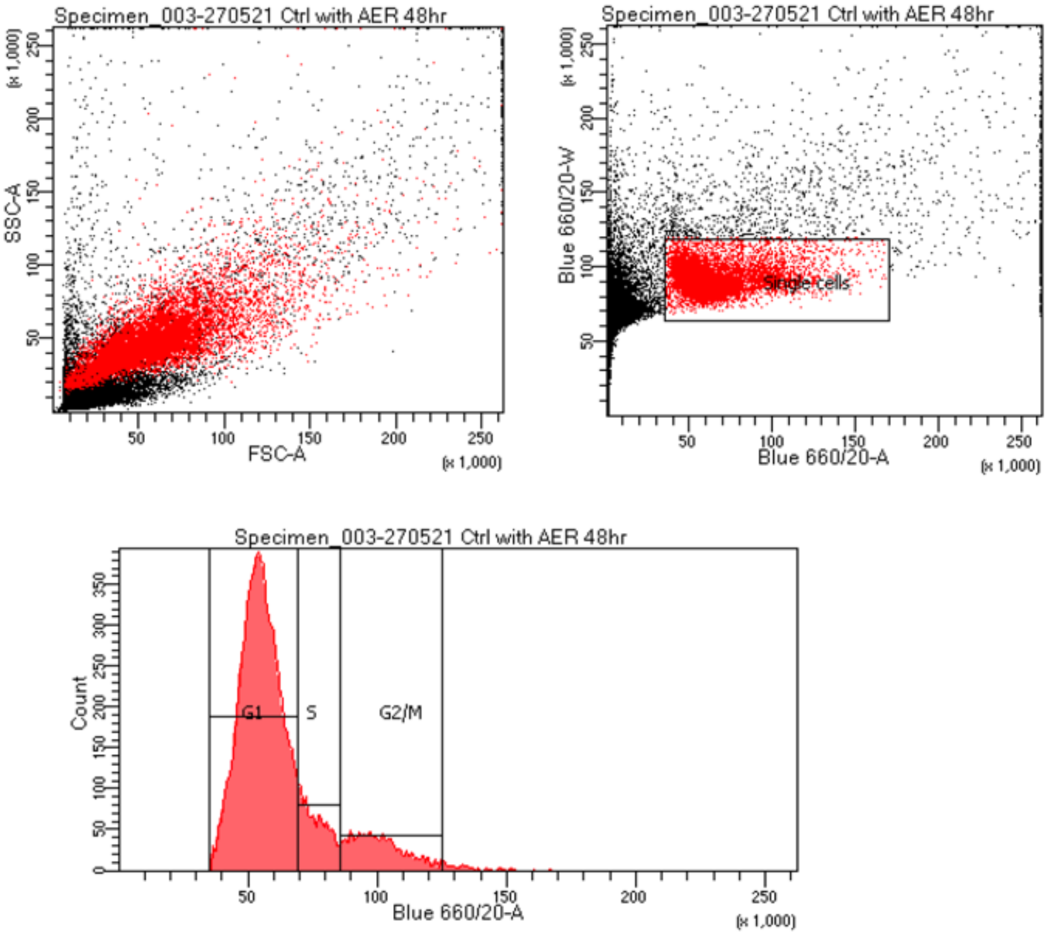

Tube: 270521 Ctrl with AER 48hr

Population	#Events	%Parent	%Total
All Events	26,868	####	100.0
Single cells	9,602	35.7	35.7
G1	7,212	75.1	26.8
S	1,010	10.5	3.8
G2/M	1,202	12.5	4.5

Figure 3a - +AER 48h – G1 = 75.84

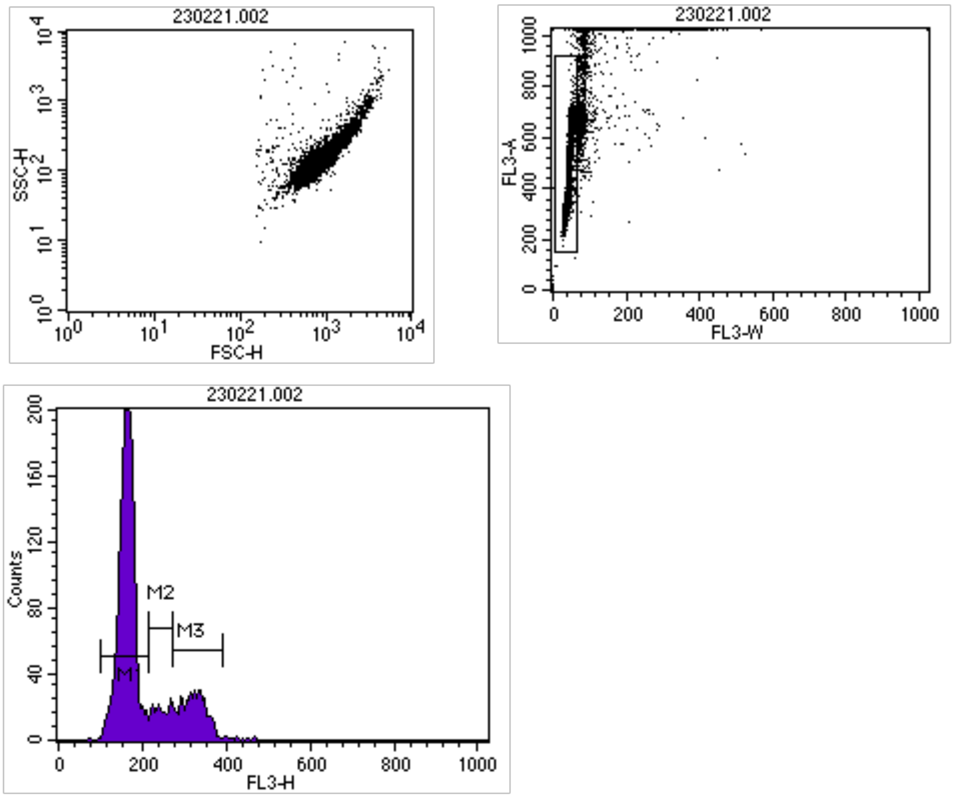

Marker	% Gated	% Total
All	100.00	87.16
M1	75.84	66.10
M2	7.01	6.11
M3	17.13	14.93

Figure 3a - No AER 24h – G1 = 69.05

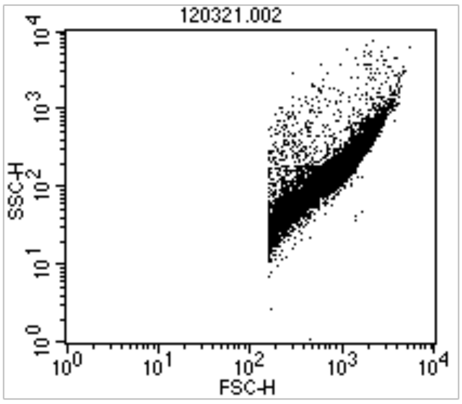

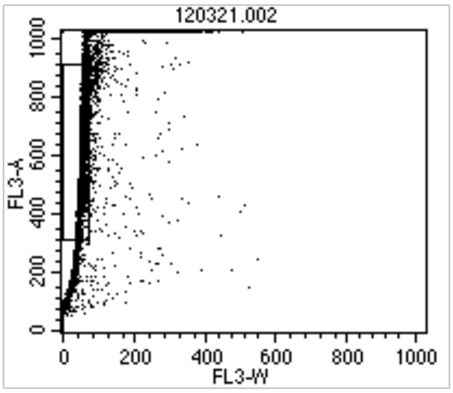

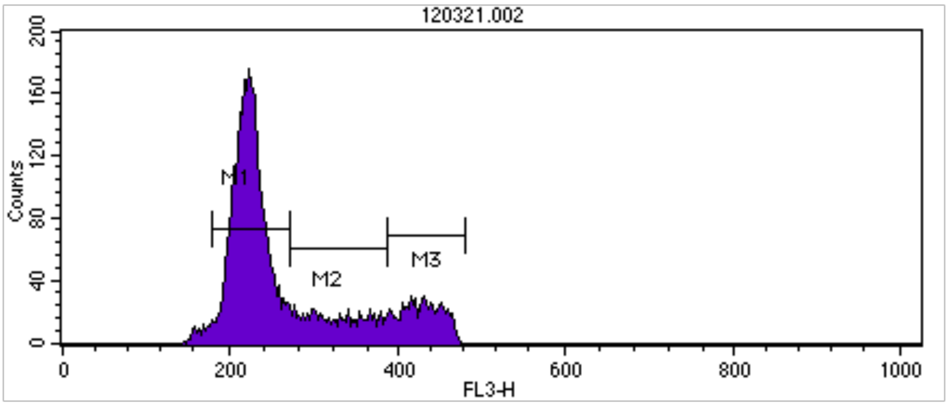

Marker	Left, Right	Events	% Gated	% Total	Mean	Geo Mean	CV	Median	Peak Ch
All	0, 1023	10000	100.00	65.21	267.74	257.81	29.59	231.00	221
M1	178, 271	6799	67.99	44.34	223.01	222.30	8.02	222.00	221
M2	271, 388	1584	15.84	10.33	325.60	323.72	10.77	323.00	271
M3	388, 481	1464	14.64	9.55	427.18	426.62	5.13	427.00	430

Figure 3a - No AER 24h – G1 = 68.28

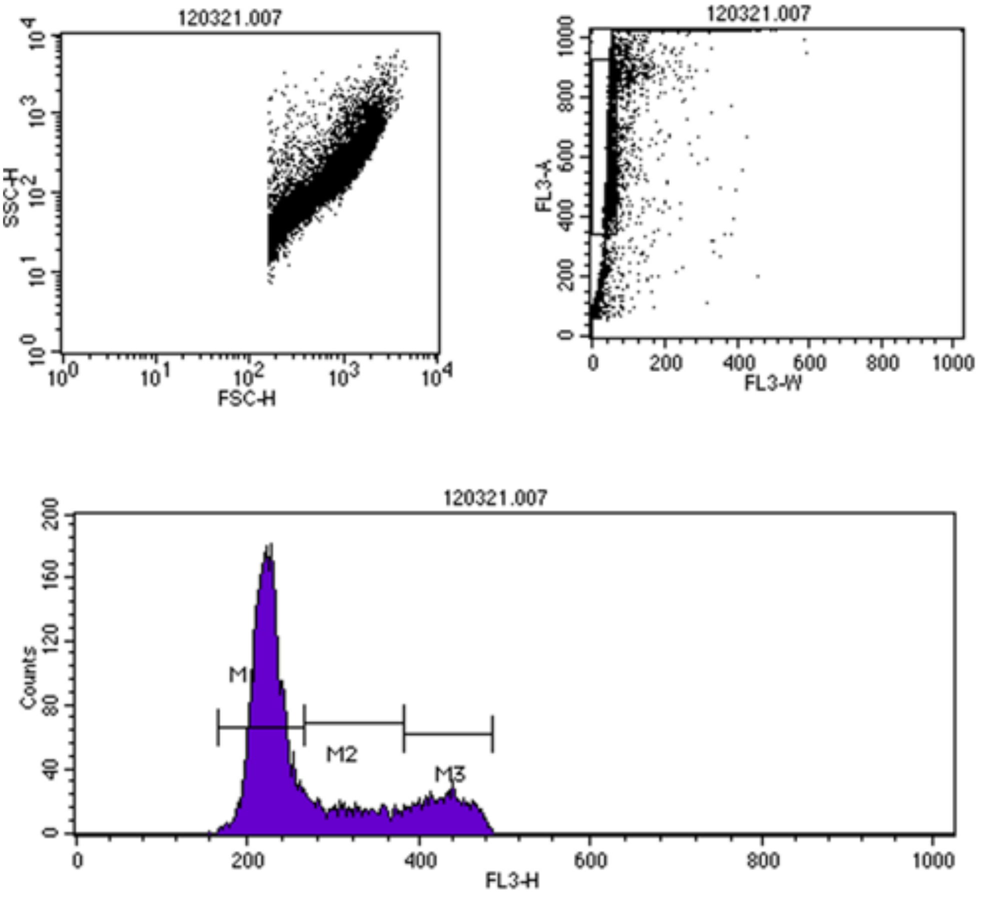

Marker	Left, Right	Events	% Gated	% Total	Mean	Geo Mean	CV	Median	Peak Ch
All	0, 1023	9580	100.00	62.80	271.16	261.28	29.50	233.00	227
M1	165, 266	6570	68.58	43.07	223.46	222.84	7.43	223.00	227
M2	266, 382	1493	15.58	9.79	318.06	316.16	10.96	316.00	266
M3	382, 486	1559	16.27	10.22	428.55	427.79	5.95	429.00	437

Figure 3a - No AER 24h – G1 = 69.09

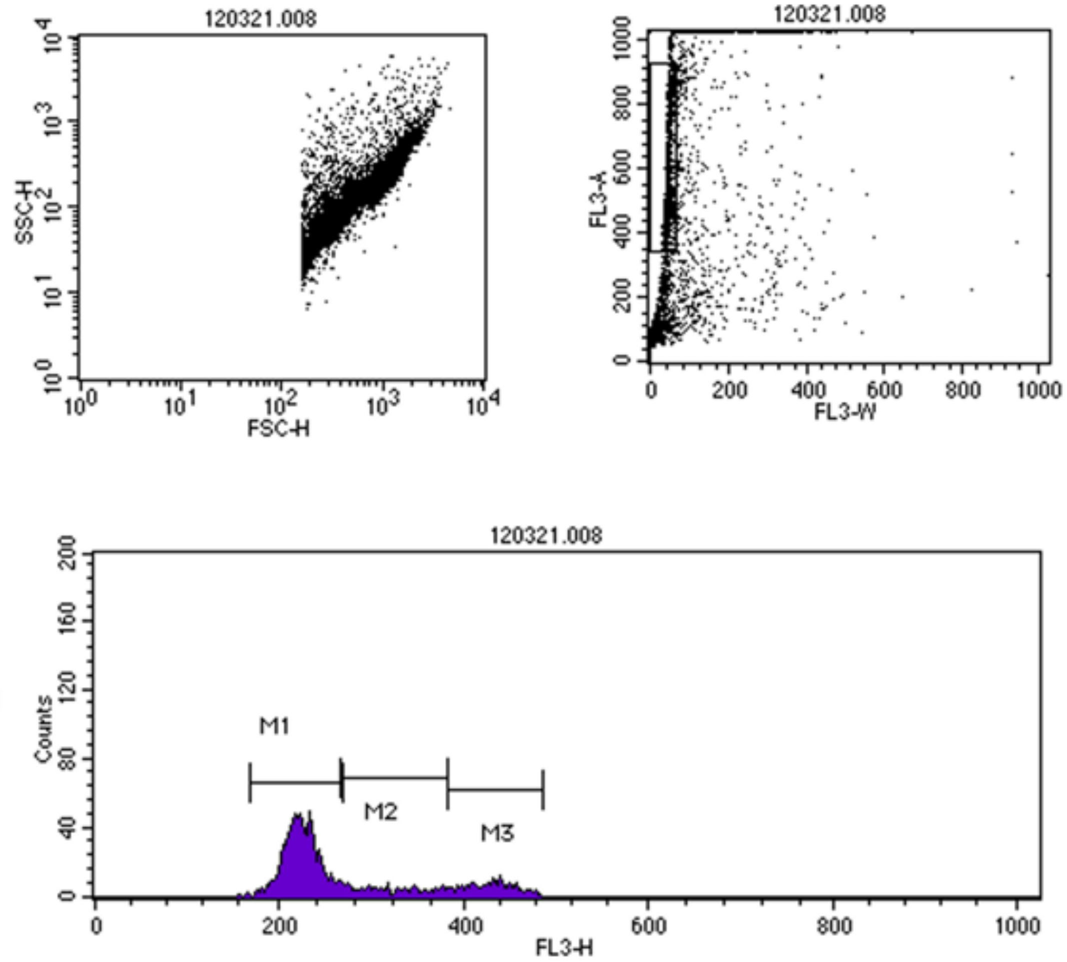

Marker	Left, Right	Events	% Gated	% Total	Mean	Geo Mean	CV	Median	Peak Ch
All	0, 1023	2489	100.00	32.60	270.68	260.71	29.62	234.00	232
M1	168, 269	1732	69.59	22.69	224.30	223.58	8.03	223.00	232
M2	266, 382	366	14.70	4.79	317.80	315.82	11.22	313.50	266
M3	382, 486	409	16.43	5.36	427.97	427.28	5.67	429.00	437

Figure 3a - No AER 48h – G1 = 82.76

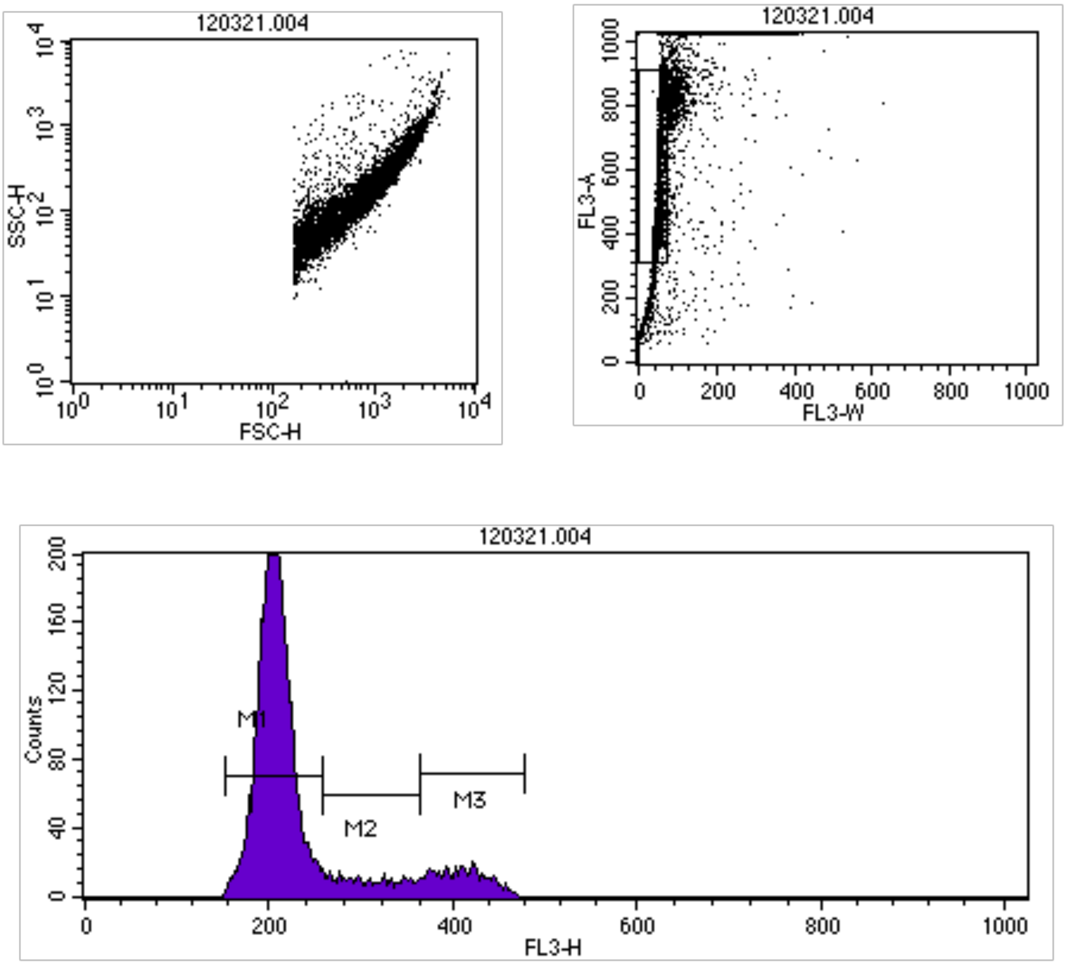

Marker	Left, Right	Events	% Gated	% Total	Mean	Geo Mean	CV	Median	Peak Ch
All	0, 1023	10000	100.00	74.26	233.54	226.40	28.25	210.00	201
M1	152, 258	8294	82.94	61.59	206.49	205.75	8.51	206.00	201
M2	258, 364	760	7.60	5.64	308.51	306.84	10.43	308.00	258
M3	364, 478	968	9.68	7.19	407.65	406.86	6.22	407.00	421

Figure 3a - No AER 48h – G1 = 88.03

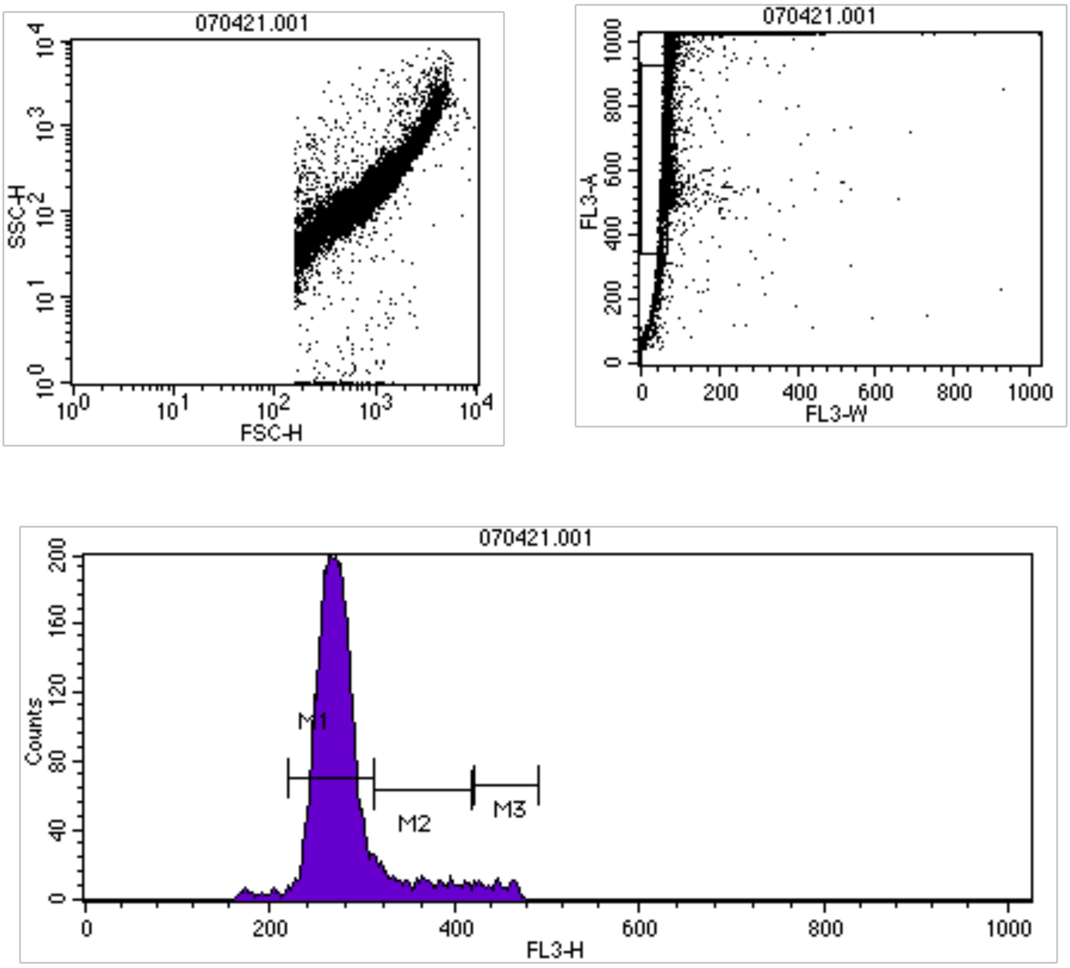

Marker	Left, Right	Events	% Gated	% Total	Mean	Geo Mean	CV	Median	Peak Ch
All	0, 1023	10000	100.00	49.62	281.33	278.53	15.30	272.00	267
M1	220, 313	8718	87.18	43.26	269.35	268.83	6.20	269.00	267
M2	313, 419	903	9.03	4.48	356.25	354.81	9.07	351.00	313
M3	421, 491	282	2.82	1.40	445.63	445.40	3.27	446.00	446

Figure 3a - No AER 48h – G1 = 73.68

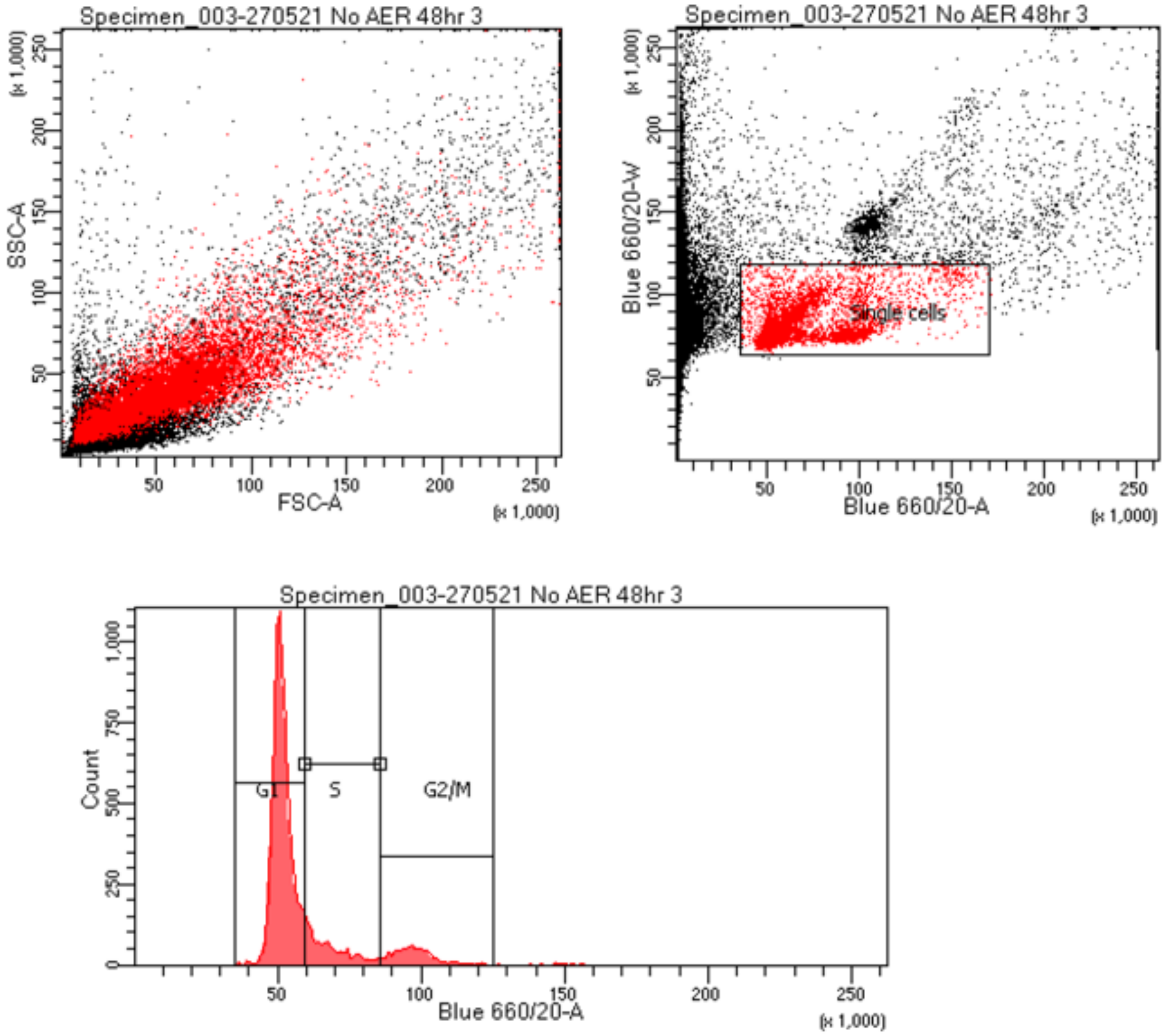

Tube: 270521 No AER 48hr 3

Population	#Events	%Parent	%Total
All Events	55,239	####	100.0
Single cells	9,990	18.1	18.1
G1	7,163	71.7	13.0
S	1,389	13.9	2.5
G2/M	1,170	11.7	2.1
