# Supplementary material for: Fgf signalling triggers an intrinsic mesodermal timer that determines the duration of limb patterning
Source: Nat Commun. 2023 Sep 20;14:5841. doi: 10.1038/s41467-023-41457-6 (PMC10511490; doi:10.1038/s41467-023-41457-6)
Supplement: Supplementary file 10 — Reporting Summary [file 41467_2023_41457_MOESM10_ESM.pdf]

nature research

Corresponding author(s): Matt Towers

Last updated by author(s): 25.08.23

Reporting Summary

Nature Research wishes to improve the reproducibility of the work that we publish. This form provides structure for consistency and transparency in reporting. For further information on Nature Research policies, see our Editorial Policies and the Editorial Policy Checklist.

Statistics

For all statistical analyses, confirm that the following items are present in the figure legend, table legend, main text, or Methods section.

n/a	Confirmed	
☐	☒	The exact sample size (*n*) for each experimental group/condition, given as a discrete number and unit of measurement
☐	☒	A statement on whether measurements were taken from distinct samples or whether the same sample was measured repeatedly
☐	☒	The statistical test(s) used AND whether they are one- or two-sided<br>*Only common tests should be described solely by name; describe more complex techniques in the Methods section.*
☒	☐	A description of all covariates tested
☐	☒	A description of any assumptions or corrections, such as tests of normality and adjustment for multiple comparisons
☐	☒	A full description of the statistical parameters including central tendency (e.g. means) or other basic estimates (e.g. regression coefficient) AND variation (e.g. standard deviation) or associated estimates of uncertainty (e.g. confidence intervals)
☐	☒	For null hypothesis testing, the test statistic (e.g. *F*, *t*, *r*) with confidence intervals, effect sizes, degrees of freedom and *P* value noted<br>*Give P values as exact values whenever suitable.*
☒	☐	For Bayesian analysis, information on the choice of priors and Markov chain Monte Carlo settings
☒	☐	For hierarchical and complex designs, identification of the appropriate level for tests and full reporting of outcomes
☒	☐	Estimates of effect sizes (e.g. Cohen's *d*, Pearson's *r*), indicating how they were calculated

Our web collection on statistics for biologists contains articles on many of the points above.

Software and code

Policy information about availability of computer code

Data collection	Zeiss Z1 Lightsheet Microscope with Zen Black 2014 SP1 Software (Zeiss), Zeiss Apotome 2 microscope with Axiovision software (Zeiss), Leica MZ16F microscope with LAS X 1.1.0.12420 software, BD Cellquest Pro for flow cytometry, Illumina HiSeq 2000 PE50 (graft RNA sequencing) and Illumina NovaSeq 6000 PE150 (Explant RNA sequencing).
Data analysis	Images were processed with Adobe Photoshop 2020 and ImageJ (Fiji) 2.14.0. Graphs and statistics with Graphpad Prism 9. HISAT v2.0.5, Feature Counts v1.5.0-p3 and DESeq2 v1.20.0 for analysis of Explant RNA sequencing data. HISAT v2.0.3, Voom and clValid for Graft RNA sequencing data.

For manuscripts utilizing custom algorithms or software that are central to the research but not yet described in published literature, software must be made available to editors and reviewers. We strongly encourage code deposition in a community repository (e.g. GitHub). See the Nature Research guidelines for submitting code & software for further information.

Data

Policy information about availability of data

All manuscripts must include a data availability statement. This statement should provide the following information, where applicable:

- Accession codes, unique identifiers, or web links for publicly available datasets
- A list of figures that have associated raw data
- A description of any restrictions on data availability

The datasets generated and/or analysed during the current study are publicly available.
The RNA sequencing data is publicly available in GEO for explants in GSE22344 (https://www.ncbi.nlm.nih.gov/geo/query/acc.cgi?acc=GSE223444) and for grafts in GSE232959 (https://www.ncbi.nlm.nih.gov/geo/query/acc.cgi?acc=GSE232959)

All flow cytometry data generated or analysed during this study are included in this published article (and its supplementary data files - Data S5.
All other data associated with figures in this study is included in the Source Data file.

Field-specific reporting

Please select the one below that is the best fit for your research. If you are not sure, read the appropriate sections before making your selection.

☒ Life sciences ☐ Behavioural & social sciences ☐ Ecological, evolutionary & environmental sciences

For a reference copy of the document with all sections, see nature.com/documents/nr-reporting-summary-flat.pdf

Life sciences study design

All studies must disclose on these points even when the disclosure is negative.

Sample size	Sample size was chosen based on the number of healthy, viable embryos available. Student t-tests were widely used in the study which required a minimum sample size of 3.
Data exclusions	No data excluded.
Replication	All multiplexed hybridization chain reaction (HCR), EdU labeling and Lysotracker assays were performed on over 3 biological replicate explants. All attempts at replication of these experiments were successful and we have included representative images with replicate information in the manuscript. All flow cytometry assays were performed with pooled samples of 8-12 explants with over 3 biological replicates.
Randomization	Not relevant to the study. The avian eggs were delivered to us once layed, it was not possible to chose or see the embryo until opening the egg and beginning the experiment.
Blinding	Not relevant to the study. The avian eggs were delivered to us once layed, it was not possible to chose or see the embryo until opening the egg and beginning the experiment, therefore blinding was already incorporated into the nature of the experiment.

Reporting for specific materials, systems and methods

We require information from authors about some types of materials, experimental systems and methods used in many studies. Here, indicate whether each material, system or method listed is relevant to your study. If you are not sure if a list item applies to your research, read the appropriate section before selecting a response.

Materials & experimental systems

n/a	Involved in the study	
☒	☐	Antibodies
☒	☐	Eukaryotic cell lines
☒	☐	Palaeontology and archaeology
☐	☒	Animals and other organisms
☒	☐	Human research participants
☒	☐	Clinical data
☒	☐	Dual use research of concern

Methods

n/a	Involved in the study	
☒	☐	ChIP-seq
☐	☒	Flow cytometry
☒	☐	MRI-based neuroimaging

Animals and other organisms

Policy information about studies involving animals; ARRIVE guidelines recommended for reporting animal research

Laboratory animals	Chicken - Bovans Brown embryos. The embryos were used between HH20 and HH36 as specified in the materials and methods. The sex of the embryo was not relevant to the study.
Wild animals	The study did not involve wild animals
Field-collected samples	The study did not involve field collected samples
Ethics oversight	Ethical approval not required for work on birds younger than 2/3 of the way through the incubation period

Note that full information on the approval of the study protocol must also be provided in the manuscript.

Flow Cytometry

Plots

Confirm that:

☒ The axis labels state the marker and fluorochrome used (e.g. CD4-FITC).

☒ The axis scales are clearly visible. Include numbers along axes only for bottom left plot of group (a 'group' is an analysis of identical markers).

☒ All plots are contour plots with outliers or pseudocolor plots.

☒ A numerical value for number of cells or percentage (with statistics) is provided.

Methodology

Sample preparation	Chick wing bud distal mesenchyme pooled from 8-12 replicate experiments were dissected in PBS under a LeicaMZ16F microscope using fine surgical scissors, and digested into single cell suspensions with trypsin (0.05%, Gibco) for 30 mins at room temperature. Cells were briefly washed in PBS, fixed in 70% ethanol overnight, washed in PBS and re-suspended in PBS containing 0.1% Triton X-100, 50 µg/ml−1 of propidium iodide and 50 µg/ml−1 of RNase A (Sigma). Dissociated cells were left at room temperature for 20 mins, cell aggregates were removed by filtration and single cells analysed for DNA content with a FACSCalibur flow cytometer and FlowJo software (Tree star Inc.).
Instrument	FACSCaliber Serial number: E2519
Software	Flow cytometry data was collected using BD CellQuest Pro software and statistical analysis was performed using GraphPad Prism.
Cell population abundance	Single cells were obtained by trypsinising the samples until a single cell suspension was achieved, this was then purified by filtering out cells which had been stuck together. Gating using forward scatter was then used to measure only the single cell population. The gate was predetermined to take 10,000 cells for each sample repeat.
Gating strategy	Single cells were gated using the forward scatter to determine which cells were doublet cells and therefore should not be included in the gate. The chosen population of cells were then divided into 3 populations based on DNA content. This is indicated by the amount of propidium iodide signal (arbitary units) and thus, peaks. Raw data is included in supplementary data 5 showing the gates.

☒ Tick this box to confirm that a figure exemplifying the gating strategy is provided in the Supplementary Information.
